# Effects of Wearable Fitness Trackers and Activity Adequacy Mindsets on Affect, Behavior, and Health: Longitudinal Randomized Controlled Trial

**DOI:** 10.2196/40529

**Published:** 2023-01-25

**Authors:** Octavia Hedwig Zahrt, Kristopher Evans, Elizabeth Murnane, Erik Santoro, Michael Baiocchi, James Landay, Scott Delp, Alia Crum

**Affiliations:** 1 Department of Organizational Behavior Stanford University Graduate School of Business Stanford, CA United States; 2 Department of Psychology Stanford University Stanford, CA United States; 3 Department of Computer Science Stanford University Stanford, CA United States; 4 Department of Epidemiology and Population Health Stanford University Stanford, CA United States; 5 Department of Mechanical Engineering, Department of Bioengineering Stanford University Stanford, CA United States

**Keywords:** physical activity, health technology, psychology, mindset, mobile health, mHealth, activity trackers, fitness trackers, activity monitors, wearables, health behavior, digital health, health promotion, intervention, mobile phone

## Abstract

**Background:**

There is some initial evidence suggesting that mindsets about the adequacy and health consequences of one’s physical activity (*activity adequacy mindsets [AAMs]*) can shape physical activity behavior, health, and well-being. However, it is unknown how to leverage these mindsets using wearable technology and other interventions.

**Objective:**

This research examined how wearable fitness trackers and meta-mindset interventions influence AAMs, affect, behavior, and health.

**Methods:**

A total of 162 community-dwelling adults were recruited via flyers and web-based platforms (ie, Craigslist and Nextdoor; final sample size after attrition or exclusion of 45 participants). Participants received an Apple Watch (Apple Inc) to wear for 5 weeks, which was equipped with an app that recorded step count and could display a (potentially manipulated) step count on the watch face. After a baseline week of receiving no feedback about step count, participants were randomly assigned to 1 of 4 experimental groups: they received either accurate step count (reference group; 41/162, 25.3%), 40% deflated step count (40/162, 24.7%), 40% inflated step count (40/162, 24.7%), or accurate step count+a web-based meta-mindset intervention teaching participants the value of adopting more positive AAMs (41/162, 25.3%). Participants were blinded to the condition. Outcome measures were taken in the laboratory by an experimenter at the beginning and end of participation and via web-based surveys in between. Longitudinal analysis examined changes within the accurate step count condition from baseline to treatment and compared them with changes in the deflated step count, inflated step count, and meta-mindset conditions.

**Results:**

Participants receiving accurate step counts perceived their activity as more adequate and healthier, adopted a healthier diet, and experienced improved mental health (Patient-Reported Outcomes Measurement Information System [PROMIS]-29) and aerobic capacity but also reduced functional health (PROMIS-29; compared with their no-step-count baseline). Participants exposed to deflated step counts perceived their activity as more inadequate; ate more unhealthily; and experienced more negative affect, reduced self-esteem and mental health, and increased blood pressure and heart rate (compared with participants receiving accurate step counts). Inflated step counts did not change AAM or most other outcomes (compared with accurate step counts). Participants receiving the meta-mindset intervention experienced improved AAM, affect, functional health, and self-reported physical activity (compared with participants receiving accurate step counts only). Actual step count did not change in either condition.

**Conclusions:**

AAMs––induced by trackers or adopted deliberately––can influence affect, behavior, and health independently of actual physical activity.

**Trial Registration:**

ClinicalTrials.gov NCT03939572; https://www.clinicaltrials.gov/ct2/show/NCT03939572

## Introduction

### Background

Most people now know that physical activity is vital for health. Over the past few decades, if one has seen a physician, read the news, or obtained brochures from their health insurance or flyers from a local gym, they are likely aware of this fact. Indeed, we are constantly reminded of the need to engage in adequate physical activity. Media and public health messages frequently assert that “Regular physical activity is one of the most important things people can do to improve their health” [1]. News headlines warn us about the “Ways a Sedentary Lifestyle Is Killing You”––and that “Sitting Is the New Smoking” [2]. There is even scientific evidence suggesting that the “lack of exercise [is] responsible for twice as many deaths as obesity” [3].

However, to many of us, it is less clear how exactly to engage in an “adequate” amount of physical activity and whether we are doing enough. Guidelines on the amounts and types of physical activity needed to promote one’s health have shifted over time [1,4] based on growing scientific evidence and debates about how to distill research findings into simple recommendations [5]. Fitness trackers often recommend walking 10,000 steps per day [1,6], but this number has been criticized as arbitrary or too high [7]. Perhaps what is worse is that physical activity recommendations are so ambitious that they can seem out of reach, with 76% of American adults reportedly not meeting them [8]. This may leave many of them believing that their level of physical activity is inadequate and that their health is suffering as a result. However, do these beliefs––or mindsets—about the adequacy of one’s physical activity matter? Could they undermine our health and well-being, perhaps even independently of how much physical activity we are actually engaging in?

A large body of research has explored how to promote higher levels of physical activity, for example, by leveraging wearable activity trackers and smartphone apps [9-15] or behavior change techniques (BCTs) [16]. However, this literature has largely overlooked the potential role of psychological mindsets, which might help explain the mixed evidence on the effectiveness of behavioral interventions. Our research sought to fill this gap. First, we reviewed the theory and evidence of the effects of *activity adequacy mindsets (AAMs)* on behavior, health, and well-being. We then proposed factors that might shape individuals’ AAMs, including wearable fitness trackers. Next, we asked how we might harness these mindsets to improve health and well-being at scale. Finally, we conducted a longitudinal field experiment exploring these questions empirically.

### Mindsets and Their Effects on Health and Well-being

Mindsets are our core assumptions regarding a domain or category (eg, intelligence, healthy eating, stress, and physical activity) [17,18]. They help us organize, simplify, and interpret information, thereby orienting us toward a particular set of expectations, attributions, and goals. Mindsets predispose us toward a particular way of experiencing and responding to situations. Owing to the complexity and ambiguity of life, people can have very different mindsets about aspects of themselves and the world––and these mindsets can have substantial consequences.

Decades of psychological research show that mindsets are critical yet often overlooked factors influencing individuals’ motivation, behavior, and performance (eg, mindsets about intelligence) [19]. More recently, an emerging body of research suggests that mindsets about aspects of health-relevant behaviors and processes––such as stress [17,20], diet [21,22], and aging [23]––can shape their effects on health and well-being.

In the context of physical activity, initial research suggests that people have mindsets about their physical activity level’s adequacy and its corresponding health consequences (*AAMs*) [24]. These mindsets are based *partly* on individuals’ actual physical activity. However, they are often not a mere reflection of one’s objective activity levels. For example, even among individuals who engage in the same objective amount of physical activity, some may believe that their activity level is adequate and benefits their health (ie, *adequate* activity mindset). In contrast, others may believe that their activity is inadequate and harms their health (ie, *inadequate* activity mindset). Individuals’ AAMs may substantially affect their health, well-being, and even longevity regardless of their actual physical activity. In epidemiological research, data from 3 nationally representative samples showed that people who perceived themselves as less active than other people their age (a proxy for the inadequate activity mindset) had a mortality risk up to 72% higher 21 years later than those who perceived themselves as more active, controlling for actual amounts of activity (assessed through comprehensive self-report questionnaires and objective accelerometer data) [25]. Similarly, perceived physical activity relative to others predicts cognitive function in older adults [26], and perceived sedentary behavior relative to others is associated with psychological stress [27].

In experimental research, a study examined a sample of hotel room attendants who objectively met physical activity guidelines through their work but still perceived themselves as inactive as they were unaware that their work counted as exercise. An intervention informing room attendants that their work constituted adequate exercise resulted in reduced weight, body fat, and blood pressure 1 month later compared with a control group [28]. Another study [24] investigated the effects on AAM of viewing the official US physical activity guidelines (prescribing a relatively high amount of activity) compared with guidelines that prescribed a lower amount of activity. Individuals exposed to guidelines prescribing a lower amount of physical activity adopted more adequate activity mindsets, which in turn predicted greater self-efficacy, engagement in physical activity, and perceived health 1 week later. Moreover, a meta-analysis comparing the effects of exercise training and placebo exercise training (ie, types of exercise without a known pharmacological, biochemical, or physical mechanism of action) showed that the mere belief that one is engaging in exercise accounted for half of the psychological benefits of exercise (eg, reduced anxiety and depression) [29]. Presumably, these effects occurred because participants had adequate activity mindsets and, thus, expected well-being benefits.

Although these studies provide suggestive evidence that AAMs may affect health and well-being, other studies have yielded less promising results. In particular, one intervention failed to induce positive changes in mindsets about physical activity [30], and another was unable to produce effects on health outcomes in healthy adolescents [31]. Moreover, research and public attention on the important effects of actual physical activity behavior on health has continued to predominate [1] at the expense of the insight that mindsets may also matter.

**Figure 1:**
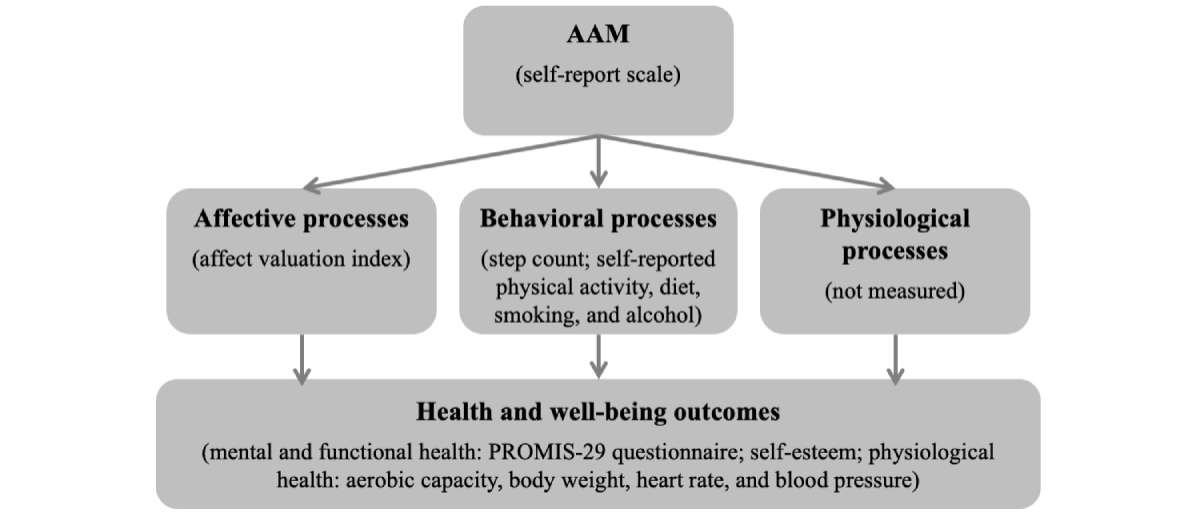
Conceptual model of how activity adequacy mindset (AAM) may influence health and well-being through affective, behavioral, and physiological processes and how these are measured in this study.

To better understand when and why AAMs can influence health and well-being, it is important to explain *how* mindsets may work. Psychological and medical research suggests that mindsets may work through affective, behavioral, and physiological processes (Figure 1). First, a person’s AAM may influence their *affective experiences*. For example, the mindset that one’s physical activity level is inadequate is associated with negative affect. It may increase health-related stress and anxiety as physical inactivity is widely known to be a substantial threat to health [32]. Affective experiences, in turn, are strong predictors of mental health and well-being [33], physical health, and longevity [34]. Affect is associated with a range of psychobiological processes implicated in most major diseases [35], including activation of the inflammatory response system and the hypothalamic-pituitary-adrenal axis, which regulate the stress response and the metabolic, cardiovascular, immune, and central nervous systems [36-38].

Second, a person’s AAM may affect their *engagement in healthy behaviors*. On the one hand, an adequate activity mindset may reduce the effort spent on physical activity by implying that the current state is close to the goal state [39]. On the other hand, an adequate activity mindset and its associated positive affect may serve as a reward for continued engagement. It may also increase commitment to a healthy lifestyle [40] and lead individuals to adopt a “healthy identity” [41], which may motivate them to adopt a range of healthy behaviors in addition to exercise, such as eating a wholesome diet.

Third, a person’s AAM may induce *physiological processes* underpinning the placebo effect, a robust and well-documented phenomenon. Placebo effects are physiological and psychological responses to drugs and treatments that are not caused by any active ingredients but by individuals’ expectations that they will produce particular effects [42-44]. Placebo effects can induce clinically significant changes in various conditions, including pain, allergies, hypertension, and Parkinson disease. They do so via specific neurobiological mechanisms, including the activation of the neuronal, cardiovascular, and endocrine systems. For example, a study showed that the effect of remifentanil, a potent opioid analgesic, depends on individuals’ expectations that the drug will reduce pain—positive treatment expectations doubled the drug’s effect, whereas negative treatment expectations eliminated the pain-relieving effects. In addition, subjective pain was underpinned by activity in pain-regulating brain regions [45]. In summary, AAMs may influence health and well-being through known affective, behavioral, and physiological pathways. However, we need more research to fully understand psychophysiological links; reconcile mixed results from past studies; and examine factors shaping AAMs, such as the increasingly ubiquitous modern personal health technologies.

### Can Wearable Technology Shape AAMs?

Individuals’ AAMs do not simply reflect their objective physical activity levels; indeed, a study found a moderate correlation of 0.32 between actual and perceived activity levels [25]. Instead, activity mindsets are susceptible to external sources of information such as exercise guidelines [24] and social comparison [25-27]. Wearable technology may be another influential source of AAMs.

The basic idea behind wearable activity trackers is simple: help users engage in adequate physical activity by providing feedback about their progress toward specific activity goals (eg, 10,000 steps a day, standing for at least 1 minute per hour during 12 hours of the day, or exercising for a target number of minutes). Much research attention has been devoted to eHealth and mobile health interventions to promote physical activity [15], and commercial high-end wearables now also incorporate other BCTs such as highlighting the discrepancy between current behavior and goal, biofeedback, social comparison, and social support [16]. Unfortunately, the evidence of activity trackers’ effectiveness is inconclusive. Some meta-analyses have found improvements in physical activity [9] and body weight [12], but others have shown no or even pernicious effects when comparing wearable-based interventions with alternative interventions (rather than inactive controls) [10,11,46]. A reason for these inconsistent and sometimes adverse effects may be that trackers may have unintended effects on AAMs. When individuals see that their step count is low or does not meet the target, they may adopt the mindset that their activity level is inadequate and, thus, harmful to their health. This effect may be particularly pronounced as trackers make users’ activity levels chronically salient. Even so, previous research does not allow us to cleanly examine the effects of the *mindset* that a user forms based on a tracker given that the effects of this mindset are confounded by the effects of the *actual physical activity level* displayed by the tracker.

### How Can We Harness Mindsets to Promote Health and Well-being at Scale?

AAMs have the potential to improve health and well-being, but to date, interventions to leverage AAMs at scale are lacking. First, to establish causal effects, research has used deceptive methods to manipulate mindsets [24], which would be unethical outside the research context. Second, a high level of specificity makes interventions challenging to scale. For example, the intervention informing room attendants that their work satisfies exercise guidelines [28] cannot be adapted to less physically demanding jobs and neglects other aspects of a person’s lifestyle (eg, carrying children). Third, interventions explicitly teaching participants to adopt a mindset (rather than inducing it stealthily) have shared information about the *content* of the desirable mindset (eg, “my work is good exercise”) but not about *mindsets per se* (eg, “assuming that my physical activity is inadequate is a mindset that is not necessarily true”) or their effects (eg, “my mindsets can influence my health and performance”). Interventions lacking such meta-cognitive knowledge about mindsets may be less likely to stick in the long run when individuals’ lifestyles change or their environments present inconsistent information.

An alternative approach is to share scientific insights about the power of mindsets with individuals directly and teach them strategies to harness mindsets in their own lives. This *meta-mindset* intervention approach [47,48] allows individuals to adopt a meta-cognitive perspective on mindsets by encouraging them to become aware of and question their current mindsets. Meta-mindset interventions also teach skills and strategies (such as monitoring, reflection, and planning) that empower people to consciously adopt useful mindsets and apply insights to various life domains and situations. There is initial evidence of the effectiveness of meta-mindset interventions. In one study, employees were taught that stress can have both debilitating and enhancing effects and that stress mindsets can influence the effects of stress in a self-fulfilling manner. This intervention enabled participants to adopt a stress-is-enhancing mindset and improve their physical health, health satisfaction, and work performance [47]. Another intervention successfully encouraged students to deliberately adopt a growth mindset about intelligence and an internal locus of control [48]. Similarly, learning about AAMs and their effects may empower individuals to choose beneficial mindsets and improve their health and well-being.

### This Research

This research explored 4 questions arising from the theory and evidence reviewed previously. First, we examined whether receiving step count feedback from a wearable tracker (in this case, Apple Watch [Apple Inc]) affects one’s AAM. Second, we experimentally manipulated step count feedback with the intent of inducing different levels of AAM and thereby investigate whether AAMs causally influence health and well-being (eg, weight and blood pressure, anxiety and depression, and ability to engage in everyday tasks) independently of how active individuals actually are. Third, we explored AAMs’ effects on several affective and behavioral determinants of health (eg, positive and negative affective experiences, physical activity, and diet). Fourth, we tested the effectiveness of a meta-mindset intervention designed to empower individuals to deliberately adopt AAMs that can benefit their health and well-being.

## Methods

### Overview

This study was preregistered on ClinicalTrials.gov [49]. Instead of confirming a small set of hypotheses, this research aimed to uncover novel insights and generate further hypotheses in an exploratory way. Therefore, a range of measures was collected and analyzed. In considering the results, the focus should be on consistent patterns of findings rather than statistical significance levels (which were not adjusted for multiple testing; see the study by Rubin [50]), and all results may be considered preliminary. The complete methods and results are included in Multimedia Appendix 1 [51-56].

### Participants and Procedures

Participants were a diverse sample of 162 West Coast community-dwelling adults recruited via flyers and web-based platforms (ie, Craigslist and Nextdoor) between September 2017 and September 2019. The posting advertised an opportunity to participate in a paid research study to develop more effective fitness trackers. To be eligible to participate, they had to meet the following criteria assessed via a web-based prescreening survey: walking as the primary source of physical activity in the previous 6 months (to ensure relevance of the step count manipulation), health status allowing for engagement in physical activity according to the Physical Activity Readiness Questionnaire [51], not being pregnant (as natural changes in weight and body composition during pregnancy would invalidate the results), possession of an iPhone 5s or newer (to allow for connection to an Apple Watch), and limited exposure to activity-tracking technology or apps (to ensure that participants were naïve to their daily step count).

**Figure 2:**
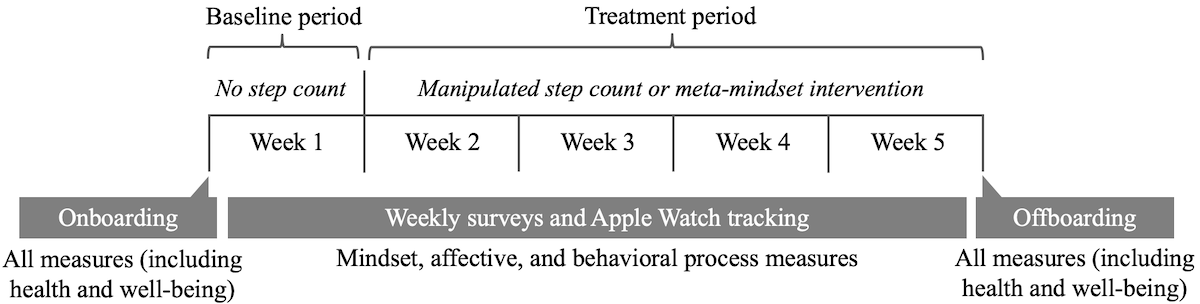
Study timeline.

Each participant attended a personal onboarding and offboarding session in a laboratory of the Computer Science department at Stanford University at the start and end of their 5-week study participation (Figure 2). In the onboarding session, participants received an Apple Watch Series 1 equipped with “AccuSteps,” a step-tracking app developed by the research team that can collect and manipulate a user’s step count and ambiently displays that information as a widget on the watch face. Participants were briefed with the cover story that the study aimed to develop more accurate fitness-tracking algorithms. They then provided informed consent and received a handout explaining the benefits of walking for health and well-being, anchoring them on the idea that every additional step is valuable even at low physical activity levels. Participants were also instructed to use only the AccuSteps app for physical activity information and to wear the Apple Watch every day (except when sleeping, showering, or swimming). They then completed web-based psychological assessments, and the experimenter performed physiological assessments.

Throughout the following 5 weeks, participants’ step counts were tracked using the Apple Watch. In addition, participants completed weekly web-based surveys assessing affective and behavioral processes and daily web-based check-ins to ensure step count awareness. A researcher monitored participants’ survey response rates and watch activity to ensure study adherence. When step counts had not been uploaded to the cloud database for an extended time, researchers communicated with participants via SMS text message or email to remind them to wear the watch or assist with any technical issues. At the end of the 5 weeks, participants returned for the offboarding session, completing the same measures as in the onboarding session. They were then fully debriefed, thanked, and paid US $175 for satisfactory participation. Suspicions regarding the experimental manipulations and study purpose were probed with up to 10 progressively specific questions during poststudy interviews. The degree of suspicion was then rated by the researcher (1=not at all, 2=slightly, 3=somewhat, 4=very, and 5=extremely).

A total of 45 participants were excluded from the analysis because of incomplete study participation (n=30, 67%), substantial suspicion about experimental manipulations (ie, correctly guessing inflation or deflation of step count, with suspicion rating >3; n=4, 9%), noncompliance (ie, using other activity-tracking apps or comparing step counts with friends; n=9, 20%, including 7, 16%, who also expressed substantial suspicion), or pregnancy discovered during the study period (n=2, 4%), yielding the final sample size of 162.

Further details on the methods are included in Multimedia Appendix 1.

### Ethics Approval

This study was approved by the Stanford University institutional review board (protocol 36098).

### Design and Manipulations

#### Overview

This study used a parallel trial design (allocation ratio 1:1:1:1). Participants were assigned to one of four conditions––(1) accurate step count (41/162, 25.3%), (2) deflated step count (40/162, 24.7%), (3) inflated step count (40/162, 24.7%), or (4) meta-mindset intervention plus accurate step count (41/162, 25.3%)––via criteria-based randomization, a novel procedure that helps minimize imbalances in premanipulation covariate distributions across experimental groups to increase precision and statistical power [52,53]. Week 1 was the baseline period, during which no step count feedback or interventions were delivered. Weeks 2 to 5 were the treatment period. The meta-mindset intervention was delivered on day 7, and the Apple Watch step count was displayed to all participants starting on day 8. The participants and the experimenter interacting with them were blind to the condition. Participants were also unaware that there were any experimental conditions.

#### Step Count Feedback Manipulations

**Figure 3:**
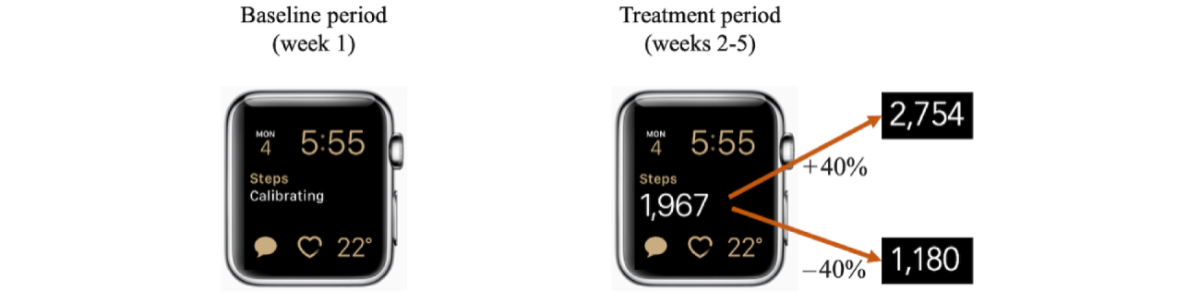
Illustration of an Apple Watch (Apple Inc) with the AccuSteps app displaying the manipulated step count.

After the no-feedback baseline period (week 1), participants in the accurate step count condition started to view their step count as recorded by the Apple Watch (Figure 3). This condition allowed us to examine whether simply wearing an activity tracker and receiving step count feedback (vs no feedback) was associated with changes in AAM and other outcomes. After the baseline period, participants in the deflated and inflated step count conditions started to view their step count as recorded by the Apple Watch but automatically deflated or inflated by 40%, respectively, by our AccuSteps app. Participants in the meta-mindset condition received an accurate step count. All participants believed that they were receiving their accurate step count (confirmed via poststudy interviews; see the *Participants and Procedure* section).

#### Meta-Mindset Intervention

The meta-mindset intervention was included in the first weekly survey and consisted of 3 videos and reflection activities. The 3- to 5-minute–long videos informed participants about health-related mindsets in general, AAMs in particular, and how mindsets can create self-fulfilling effects. The reflection activity prompted participants to *notice* any activities that they had performed in the last week that required some physical effort (eg, walking, housework, and other activities they might not usually think of as exercise). Next, they were asked to *count* all these activities as beneficial exercise and *celebrate* themselves for this physical activity. The final component encouraged people to think about their physical activity’s short- and long-term benefits (eg, improved mood and sleep, lower blood pressure, and protection from heart disease). Participants completed a short version of this reflection activity in each subsequent daily check-in and weekly survey. See Multimedia Appendix 1 for details.

### Measures

#### Overview

All measures (ie, health outcomes, AAM, and affective and behavioral measures) were measured in the laboratory at onboarding and offboarding. In addition, a subset of measures (ie, AAM and affective and behavioral measures) was taken in weekly surveys. Survey scales providing reference periods referred to either the last 30 days (in onboarding or offboarding surveys) or the last 7 days (in weekly surveys). Step count was continuously tracked via the Apple Watch.

#### AAM (Manipulation Check)

A 5-item version of the Activity Adequacy Mindset Scale [24] was used (eg, “How beneficial is your current level of physical activity for your health?”; 1=*Not at all beneficial* to 5=*Extremely beneficial*; and “How much does your current level of physical (in-) activity increase or decrease your risk of disease?”; 1=*Increases my risk very much* to 7=*Decreases my risk very much*). The items were averaged into a composite score ranging from 1 to 7, with higher scores indicating a more adequate activity mindset (Cronbach α≥.85).

#### Health Outcomes

*Mental health* was measured using the Patient-Reported Outcomes Measurement Information System [PROMIS]-29 profile version 2.0 [57] subscales for anxiety (4 items; eg, “In the past 30 days, I found it hard to focus on anything other than my anxiety”; 1=*Never* to 5=*Always*), depressive symptoms (4 items; eg, “In the past 30 days, I felt worthless”; 1=*Never* to 5=*Always*), sleep disturbance (4 items; eg, “In the past 30 days, I had difficulty falling asleep”; 1=*Not at all* to 5=*Very much*), and fatigue (4 items; eg, “In the past 30 days, how fatigued were you on average?”; 1=*Not at all* to 5=*Very much*). Items were averaged into a composite score ranging from 1 to 5, with higher scores reflecting better mental health (Cronbach α=.92).

*Self-esteem*, an important indicator of well-being, was assessed via the single-item self-esteem scale [58]— “I have high self-esteem” (1=*Not very true of me* to 5=*Very true of me*).

*Functional health* was measured using the PROMIS-29 profile version 2.0 subscales for physical function (4 items; eg, “Are you able to do chores such as vacuuming or yard work?”; 1=*Unable to do* to 5=*Without any difficulty*), ability to participate in social roles and activities (4 items; eg, “I have trouble doing all of my regular leisure activities with others”; 1=*Never* to 5=*Always*), pain interference (4 items; eg, “In the past 30 days, how much did pain interfere with your day to day activities?”; 1=*Not at all* to 5=*Very much*), and pain intensity (1 item; “In the past 30 days, would you rate your pain on average?”; 0=*No pain* to 10=*Worst imaginable pain*). Items were averaged into a composite score ranging from 1 to 5, with higher scores reflecting better functional health (Cronbach α≥.90).

*Physiological health* was measured using digital health monitors, specifically body weight (in pounds), resting heart rate (HR), systolic blood pressure (SBP), and diastolic blood pressure (DBP). Blood pressure measurements were combined to calculate the mean arterial pressure (MAP; an individual’s average blood pressure during a cardiac cycle) for statistical tests according to the following formula: *MAP = (SBP + 2 × DBP)* / 3. Maximal aerobic capacity was assessed in person in the onboarding and offboarding sessions using the Canadian Home Fitness Test [54] and calculated according to the following formula: *VO^2^ max = 42.5 + 16.6 × (energy requirement) − 0.12 × (body weight) − 0.12 × (final postactivity HR) − 0.24 × (age)*.

#### Affective Processes

The Affect Valuation Index [59] actual affect subscale was used to measure how often during the past 7 or 30 days, participants felt 25 affective states (1=*never* to 5=*all the time*). In total, 2 composite scores were created by averaging all items measuring positive affective states (eg, happy, excited, and peaceful; Cronbach α≥.87) and negative affective states (eg, unhappy, nervous, and sluggish; Cronbach α≥.79).

#### Behavioral Processes

*Physical activity* was assessed using 2 methods. First, step count was continuously tracked via the Apple Watch, and the end-of-day step count was used for analysis. Second, engagement in other physical activity was assessed via a self-report measure developed for this study (adapted from measures used in the National Health and Nutrition Examination Survey), providing an approximation of energy expenditure from other physical activity in kilocalories per day (see Multimedia Appendix 1 for details).

*Other health-relevant behaviors* were also measured via self-reports. Dietary choices during the past 7 or 30 days were assessed using 4 items measuring the frequency of overeating and intake of high-fat foods, sugary foods or drinks, and healthy produce. Smoking and alcohol intake were assessed using 2 items each measuring the frequency and quantity of cigarettes smoked and alcoholic drinks consumed, which were then combined into summary measures indicating overall smoking and alcohol intake.

### Analytical Approach

The mean within-participant changes in AAM, health and well-being outcomes, and affective and behavioral processes from the baseline period to the treatment period were calculated. First, for each participant separately, all baseline measurements of a particular variable were averaged, all treatment measurements were averaged, and the resulting averages were subtracted (treatment average – baseline average) to obtain the within-participant change score. Second, within-participant change scores were averaged per condition, and 95% CIs were calculated.

In addition, a multilevel longitudinal analysis was conducted to examine changes in average levels of each variable from the baseline period to the treatment period. For each variable, 2 models were fitted. Model 1 examined changes within the accurate step count condition from the baseline period to the treatment period and compared changes in the accurate step count condition (reference group) with changes in the deflated and inflated step count conditions. Model 2 compared changes from the baseline period to the treatment period in the accurate step count condition (reference group) with the meta-mindset intervention condition. This 2-model approach was chosen to account for the slight differences in what counted as baseline period for the different interventions. The step count manipulations started on day 8, the day after the first weekly survey, so that this survey was part of the baseline period. However, the meta-mindset intervention started on day 7 as part of the first weekly survey so that this survey was already part of the treatment period. AAM and affective and behavioral processes were theorized as mechanisms explaining changes in health. Thus, only their weekly (not onboarding or offboarding) measurements were used in model 1. However, because of the timing of the meta-mindset intervention, model 2 included onboarding (the only baseline measurements of AAM, affect, diet, smoking, and alcohol consumption), weekly, and offboarding measurements for these variables.

To account for the within-subject design, we included by-participant random intercepts and random slopes for the period. For models predicting changes in health and well-being outcomes, which were measured only twice, no random slopes were included because of a lack of *df*. Huber-White robust SEs were used to account for criteria-based randomization. All models (except those predicting physical activity changes) were adjusted for 2 covariates––step count and self-reported physical activity––to test whether mindset manipulations affected outcomes independently of actual physical activity. The results were consistent in models without covariate adjustments. The complete models, results, and raw data are reported in Multimedia Appendix 1.

## Results

### Effects of (Accurate) Step Count Feedback

First, we examined whether AAM, health, affect, and behavior changed from the baseline (no step count) period to the treatment period in the accurate step count condition.

#### Changes in AAM

Receiving step count feedback was associated with significant improvements in AAM from the baseline period to the treatment period (baseline mean 3.18, SD 1.05; treatment mean 3.47, SD 1.12; *b*=0.27, SE 0.10; *t*_348_=2.671; *P=*.008). That is, becoming aware of how many steps they were walking each day caused participants to believe that their physical activity level was more adequate and healthier than they had previously realized. However, average levels of AAM remained below the scale midpoint; thus, participants were still not confident that they were engaging in a health-promoting amount of activity.

#### Health Outcomes

Receiving step count feedback was associated with significant improvements in *mental health*. Participants reported fewer mental health symptoms (including anxiety, depression, sleep disturbance, and fatigue) at offboarding than at onboarding (baseline mean 3.75, SD 0.60, treatment mean 3.90, SD 0.58; *b*=0.15, SE 0.07; *t*_135_=2.139; *P=*.03). There was also a marginally significant increase in *self-esteem* (baseline mean 3.83, SD 0.74; treatment mean 3.93, SD 0.75; *b*=0.11, SE 0.06; *t*_132_=1.917; *P=*.06). In addition, the results suggested small potential improvements in *physiological health* as aerobic capacity increased from onboarding to offboarding (baseline mean 31.79, SD 8.25; treatment mean 32.34, SD 8.72); however, this increase did not reach statistical significance at the .05 level (*b*=0.56, SE 0.32; *t*_114_=1.787; *P=*.08). Resting HR (*P*=.74), body weight (*P*=.78), and blood pressure (*P*=.15) did not change, and there was a slight decline in self-reported *functional health* (baseline mean 4.49, SD 0.55; treatment mean 4.37, SD 0.58; *b*=−0.12, SE 0.05; *t*_141_=−2.387; *P=*.02).

#### Affective and Behavioral Processes

No changes were detected in *affective experiences*, that is, the frequencies of positive (baseline mean 2.54, SD 0.61; treatment mean 2.50, SD 0.59) and negative (baseline mean 2.10, SD 0.55; treatment mean 2.01, SD 0.51) affective states. Interestingly, changes were not detected in *physical activity* either, neither in step count (baseline mean 6751, SD 3306; treatment mean 6981, SD 3943; *b*=124.97; SE 254.65; *t*_128_=0.491; *P*=.62) nor in other self-reported activities (baseline mean 3.87, SD 3.52; treatment mean 3.42, SD 3.74; *b*=−0.38, SE 0.51; *t*_353_=−0.738; *P*=.46). In contrast, *other health-relevant behaviors* improved significantly. During the treatment period, participants reported consuming fewer high-fat foods (baseline mean 2.98, SD 1.67; treatment mean 2.60, SD 1.65; *b*=−0.38, SE 0.16; *t*_135_=−2.391; *P=*.02) and more healthy produce (baseline mean 4.03, SD 1.54; treatment mean 4.34, SD 1.40; *b*=0.36, SE 0.15; *t*_335_=2.309; *P=*.02) compared with the baseline period. Meanwhile, they reported maintaining their sugar intake (*P*=.58), frequency of overeating (*P*=.52), alcohol consumption (*P*=.88), and smoking (*P*=.44).

### Effects of Manipulated Step Count Feedback

Next, we examined whether deflated and inflated step count feedback led to changes in outcomes over time that differed from the changes resulting from accurate step count feedback.

#### Changes in AAM

As expected, the deflated step count caused a decline in AAM (baseline mean 3.73, SD 1.17; treatment mean 3.57, SD 1.27), which was significantly different from the AAM improvement in the accurate step count condition (*b*=−0.40, SE 0.16; *t*_348_=−2.475; *P=*.01). AAM also improved slightly in the inflated step count condition (baseline mean 3.55, treatment mean 3.69), but contrary to predictions, the change was less pronounced and not significantly different from that in the accurate step count condition (*b*=−0.08, SE 0.16; *t*_349_=−0.505; *P=*.61). Given this failed manipulation and for concision, we present the remaining results for the inflated step count condition (that were not in line with predictions) in Table 1 and Multimedia Appendix 1.

**Table 1:** Mean within-participant changes by condition from the baseline period to the treatment period (with 95% CIs).^a^

Measures		Experimental condition				
		Accurate step count	Deflated step count	Inflated step count	Meta-mindset intervention + accurate step count	
**Activity adequacy mindset (manipulation check)**						
	Mindset	0.29^b^ (0.09 to 0.49)	−0.16^c^ (−0.4 to 0.09)	0.14 (−0.11 to 0.39)	0.61^b^ (0.26 to 0.95)	
**Health outcomes**						
	Mental health	0.15^c^ (0.01 to 0.3)	−0.04^c^ (−0.18 to 0.1)	0.1 (−0.05 to 0.25)	0.2 (0.05 to 0.34)	
	Self-esteem	0.1^d^ (−0.02 to 0.21)	−0.12^c^ (−0.31 to 0.06)	−0.05 (−0.25 to 0.15)	−0.02 (−0.2 to 0.15)	
	Functional health	−0.11^c^ (−0.21 to −0.02)	−0.15 (−0.29 to −0.02)	−0.05 (−0.24 to 0.13)	0.08^b^ (−0.04 to 0.19)	
	Weight	−0.04 (−0.88 to 0.79)	−0.32 (−1.22 to 0.59)	−1.29 (−3.3 to 0.73)	0.45 (−0.65 to 1.55)	
	Resting HR^e^	0.76 (−2.04 to 3.55)	3.9^c^ (1.22 to 6.58)	3.55^d^ (1.11 to 5.99)	0.94 (−1.56 to 3.44)	
	Mean arterial pressure	−2.36 (−5.6 to 0.88)	1.7^c^ (−0.54 to 3.94)	0.95^d^ (−1.35 to 3.25)	1.28^d^ (−1.04 to 3.59)	
	Aerobic capacity	0.55^d^ (−0.05 to 1.15)	0.27 (−0.36 to 0.9)	0.03 (−0.76 to 0.81)	0.21 (−0.44 to 0.87)	
**Affective and behavioral processes**						
	Positive affect	−0.04 (−0.17 to 0.09)	−0.05 (−0.19 to 0.09)	−0.13 (−0.25 to −0.02)	−0.08^c^ (−0.24 to 0.08)^e^	
	Negative affect	−0.07 (−0.17 to 0.03)	0.04^d^ (−0.07 to 0.14)	−0.06 (−0.17 to 0.05)	−0.25^d^ (−0.39 to −0.1)^f^	
	Step count	211 (−320 to 741)	0 (−546 to 545)	−275 (−796 to 246)	166 (−354 to 686)	
	Self-reported physical activity	−0.09 (−0.77 to 0.6)	−0.73 (−2.43 to 0.96)	0.19 (−1.36 to 1.74)	0.56^d^ (−0.91 to 2.03)	
	Produce intake	0.38^c^ (0.08 to 0.68)	−0.21^b^ (−0.45 to 0.04)	0.0 (−0.32 to 0.32)	0.0 (−0.33 to 0.33)^f^	
	Fat intake	−0.37^c^ (−0.71 to −0.02)	0.2^c^ (−0.14 to 0.54)	−0.25 (−0.63 to 0.13)	−0.26 (−0.64 to 0.11)^f^	
	Sugar intake	0.02 (−0.32 to 0.36)	−0.12 (−0.49 to 0.26)	−0.49 (−0.87 to −0.12)	0.03 (−0.26 to 0.33)^f^	
	Overeating	0.05 (−0.11 to 0.22)	0.12 (−0.04 to 0.27)	−0.2 (−0.5 to 0.11)	−0.2 (−0.44 to 0.05)^f^	
	Smoking	−0.84 (−2.73 to 1.05)	0.88 (−0.37 to 2.12)	−0.86 (−3.11 to 1.39)	0.93 (−3.8 to 5.66)^f^	
	Alcohol intake	−0.23 (−0.92 to 0.46)	0.04 (−0.74 to 0.81)	−1.45^c^ (−2.99 to 0.08)	−0.12 (−0.96 to 0.73)^f^	

^a^Significance levels (derived from the multilevel longitudinal models described previously) indicate (1) whether changes from the baseline period to the treatment period in the accurate step count condition are significantly different from zero and (2) whether changes from the baseline period to the treatment period in the deflated step count, inflated step count, and meta-mindset conditions are significantly different from changes in the accurate step count condition.

^b^*P<*.01.

^c^*P<*.05.

^d^*P<*.10.

^e^HR: heart rate.

^f^These mean changes in the meta-mindset condition are not directly comparable with those in the accurate step count condition, as they are based on different time points (see the *Analytical Approach* section for details).

#### Health Outcomes

The deflated step count led to slight declines in *mental health* (baseline mean 3.67, SD 0.76; treatment mean 3.63, SD 0.64) and *self-esteem* (baseline mean 3.78, SD 0.80; treatment mean 3.65, SD 0.98), which differed significantly from the improvements observed in the accurate step count condition (mental health: *b*=−0.20, SE 0.09, *t*_148_=−2.219; *P=*.03; self-esteem: *b*=−0.24, SE 0.11, *t*_142_=−2.174; *P=*.03). *Physiological health* also changed in line with predictions. Participants in the deflated step count condition experienced increases in resting blood pressure (SBP: baseline mean 121.3, SD 12.4; treatment mean 122.7, SD 11.4; DBP: baseline mean 76.5, SD 9.4; treatment mean 78.3, SD 8.9) and HR (baseline mean 71.9, SD 10.7; treatment mean 75.8, SD 12.3), which differed significantly from the changes in the accurate step count condition (MAP: *b*=3.64, SE 1.83, *t*_152_=1.995; *P=*.048; resting HR: *b*=3.54, SE 1.79, *t*_156_=1.983; *P=*.049). Body weight (*P*=.67), aerobic capacity (*P*=.44), and *functional health* (*P*=.80) did not change or differ significantly from changes in the accurate step count condition.

#### Affective and Behavioral Processes

Changes in *affective experiences* were partially consistent with predictions as participants in the deflated step count condition experienced a slight increase in negative affect that differed marginally from the decrease in the accurate step count condition (baseline mean 2.12, SD 0.52; treatment mean 2.14, SD 0.53; *b*=0.12, SE 0.07; *t*_118_=1.814; *P=*.07). Positive affect remained unchanged, similar to the accurate step count condition (*P*>.99). In addition, exposure to deflated step count did not influence *physical activity* (step count: baseline mean 7025, SD 4286; treatment mean 7046, SD 4249; self-reported physical activity: baseline mean 6.75, SD 8.82; treatment mean 5.47, SD 7.56), and there were no detectable differences between the deflated step count and accurate step count conditions (step count: *P*=.67; self-reported physical activity: *P*=.34). In contrast, we observed significant differences in *other health-relevant behaviors* in line with predictions. Specifically, participants exposed to the deflated step count began to eat more unhealthily––consuming more high-fat foods (baseline mean 2.83, SD 1.61; treatment mean 3.05, SD 1.58) and fewer servings of healthy produce (baseline mean 4.63, SD 1.35; treatment mean 4.42, SD1.44) than they had at baseline––and these changes differed significantly from the dietary improvements in the accurate step count condition (high-fat foods: *b*=0.54, SE 0.21, *t*_118_=2.505; *P=*.01; healthy produce: *b*=−0.56, SE 0.20, *t*_335_=−2.842; *P=*.005). There were no changes and no differences compared with the accurate step count condition in sugar intake (*P*=.90), overeating (*P*=.63), alcohol consumption (*P*=.97), or smoking (*P*=.21).

### Effects of the Meta-Mindset Intervention

Finally, we examined whether the meta-mindset intervention improved AAM, affect, behavior, and health outcomes by comparing changes in these outcomes from the baseline period to the treatment period in the meta-mindset condition with changes in the accurate step count condition.

#### Changes in AAM

As predicted, AAM improved in the meta-mindset condition, and this improvement was significantly greater than that in the accurate step count condition (baseline mean 3.48, SD 1.19; treatment mean 4.06, 1.13; *b*=0.57, SE 0.18; *t*_79_=3.173; *P=*.002). In other words, participants who learned about the science of AAMs––in addition to receiving accurate step count feedback––were able to consciously change their mindsets to view their physical activity as more adequate and healthier.

#### Health Outcomes

Participants in the meta-mindset condition experienced slight improvements in *functional health* (ie, they came to feel more able to engage in everyday activities without being encumbered by pain or other health challenges; baseline mean 4.44, SD 0.58; treatment mean 4.51, SD 0.52), and these improvements differed significantly from the negative changes observed with accurate step count feedback only (*b*=0.18; SE 0.07; *t*_95_=2.714; *P=*.008). In addition, participants in the meta-mindset condition experienced improvements in *mental health* (baseline mean 3.71, SD 0.71; treatment mean 3.91, SD 0.64), similar to the improvements observed in the accurate step count condition (*P=*.76). There were no significant changes in *self-esteem* and no differences compared with the accurate step count condition (*P=*.27). In terms of *physiological health*, there were no substantial changes or deviations from the accurate step count condition (resting blood pressure: *P*=.054; resting HR: *P*=.61; body weight: *P*=.48; aerobic capacity: *P*=.49).

#### Affective and Behavioral Processes

In line with predictions, the meta-mindset intervention improved *affective experiences*. There was a reduction in the frequency of negative affective states (baseline mean 2.19, SD 0.65; treatment mean 1.94, SD 0.56), which was marginally different from the change in the accurate step count condition (*b*=−0.13, SE 0.07; *t*_78_=−1.818; *P=*.07). In addition, participants in the meta-mindset condition experienced stable levels of positive affect (baseline mean 2.92, SD 0.55; treatment mean 2.84, SD 0.74), which was a significantly more beneficial outcome compared with the decline in positive affect in the accurate step count condition (*b*=0.23, SE 0.10; *t*_80_=2.314; *P=*.02). Regarding *physical activity*, step count remained stable (baseline mean 6968, SD 3867; treatment mean 7091, SD 3753; *P=*.96), and self-reported physical activity improved slightly (baseline mean 5.08, SD 7.14; treatment mean 5.25, SD 8.53), which was marginally different from the change in the accurate step count condition (*b*=1.64, SE 0.95; *t*_89_=1.728; *P=*.09). There were some improvements in *other health-relevant behaviors* (eg, high-fat food intake: baseline mean 3.02, SD 1.68; treatment mean 2.74, SD 1.56), although these changes did not differ significantly from those observed in the accurate step count condition (fat intake: *P*=.83; produce intake: *P*=.83; sugar intake: *P*=.35; overeating: *P*=.80; smoking: *P*=.84; alcohol intake: *P*=.38).

## Discussion

### Principal Findings

This 5-week longitudinal field experiment examined the effects of wearable fitness tracker feedback on AAM. In addition, it explored the effects of AAMs on health and well-being and several affective and behavioral determinants of health. Finally, this research developed and tested a transparent, scalable meta-mindset intervention that empowers individuals to adopt beneficial activity mindsets deliberately.

The results showed that simply receiving accurate step count feedback led to improvements in participants’ AAMs, helping them realize that they were engaging in more health-promoting physical activity than they had previously believed. (However, note that participants in our sample were relatively active, with an average of 7066 steps per day at baseline compared with the US average of 4774 steps [60]; therefore, the effects of receiving step count feedback may be less beneficial for the average American adult). Participants also experienced improvements in mental health (ie, symptoms of anxiety, depression, sleep disturbance, and fatigue), self-esteem, and aerobic capacity and began eating more healthily (consuming fewer high-fat foods and more healthy produce). Unexpectedly, there was also a slight decline in functional health (ie, the perceived ability to engage in everyday activities such as running errands, working, or caring for their families without being encumbered by pain or other health challenges); in the context of the other findings, this may be attributed to heightened awareness because of measurement.

Experimentally deflating participants’ step counts by 40% led them to adopt more inadequate activity mindsets. Compared with participants receiving accurate feedback, those receiving deflated feedback adopted an unhealthier diet and felt negative affect more frequently. They also experienced declines in mental health and self-esteem and increases in resting HR and blood pressure. Finally, participants who received the meta-mindset intervention (in addition to accurate step count feedback) adopted more adequate activity mindsets and had improvements in affective experiences and functional health compared with participants receiving accurate feedback only.

Interestingly, the step count displayed by the wearable tracker appeared not to influence engagement in physical activity (in line with other research showing inconsistent or null effects of wearables and smartphone apps) [10,11,14]. Providing participants (who had no or negligible previous step count–tracking experience) with accurate step count feedback did not lead to changes in step count or other physical activity. Even more surprisingly, participants who received the arguably extreme intervention of having their step count deflated by 40% (eg, believing they walked 4200 instead of 7000 steps) did not change their physical activity. Participants receiving the meta-mindset intervention reported marginal increases in physical activity other than walking, but this may reflect an increased awareness of activity owing to the intervention.

### Limitations

This research was preregistered as an exploratory study seeking to discover uncharted territory and generate novel hypotheses [49]. As such, we collected a range of measurements and conducted multiple tests (without α-level adjustment; see the study by Rubin [50]). The results should be interpreted with caution until replicated and extended by future research. In addition, it is important not to overstate the effects of AAMs on health and well-being (which were generally small in this study; Table 1), especially when comparing them with the well-studied effects of actual physical activity. However, these findings are still important and meaningful given that healthy behaviors and health and well-being indicators are difficult outcomes to change, particularly within the relatively short 5-week time frame.

### Implications

This research has important theoretical and practical implications. First, we showed that individuals’ AAMs can shape their health and well-being independently of their actual physical activity. Although previous research provided suggestive evidence that AAMs may influence health [24,25,28], we demonstrated this relationship more rigorously by directly manipulating mindset and precisely tracking actual activity throughout the 5-week duration of the experiment. This is not to say that engaging in physical activity is not important; there is robust evidence for the critical role of physical activity in promoting health and longevity. However, this study showed that *mindsets* about physical activity also matter and should be considered when designing research studies and health promotion programs.

Second, this study adds to an emerging body of research suggesting that technology design typically overlooks effects on and of health-related mindsets [61,62] (also see the study by Ahmavand et al [63]) and it highlights opportunities to integrate raw activity-tracking information with mindset interventions (in addition to BCTs [16]) to more effectively promote healthy lifestyles and well-being. As we enter the “quantified self” era, more and more aspects of life are being tracked, from fitness, insomnia, posture, and stress levels to work hours and productivity. It is essential to carefully examine how such tracking affects mindsets as this may help explain trackers’ effectiveness as well as user engagement and acceptability [13].

Third, we provide evidence for a novel meta-mindset intervention that empowers participants to deliberately adopt more positive mindsets and use them to improve health and well-being. Our findings increase the applied utility of mindset research as meta-mindset interventions are nondeceptive and ethical outside the research context. Moreover, meta-mindset interventions have the potential to be mindfully embedded in a wide range of health promotion programs––including technology applications, public health campaigns, and workplace health programs––thereby providing a cost-effective and scalable way to enhance population health. The videos and reflection activities designed for this study (included in Multimedia Appendix 1) can be reused for such applications, although their effectiveness may be increased by adapting them to each target audience (eg, older individuals, individuals with chronic health conditions, and individuals from particular socioeconomic or cultural backgrounds). The intervention designer should consider whether AAM is the most relevant variable to target or whether other barriers to physical activity should be addressed first or in addition.

### Areas for Future Research

Further research is needed to investigate the mechanisms underlying AAMs. This will advance our theoretical understanding of mindsets and facilitate the design of interventions that target the most critical pathways in a given context. We theorize that AAMs induce a range of affective, behavioral, and physiological processes, which in turn shape health and well-being (as shown by an extensive evidence base; see the *Introduction* section). Our findings that experimentally manipulated AAMs can change affective experiences, dietary behavior, and various mental and physical health outcomes provide initial evidence for future research to build on.

In addition, the relationship between AAM and actual physical activity merits further study. We did not find robust evidence that more adequate activity mindsets lead to higher physical activity levels. Perhaps mindset does not in fact shape physical activity. Alternatively, there may be counteracting forces. For example, an adequate activity mindset may *boost* activity by increasing commitment [40] *but also reduce* activity by inducing complacency [39]. Our findings also suggest that AAMs may promote healthy behaviors other than physical activity (eg, diet), perhaps by motivating actions consistent with one’s “healthy identity” [41].

Moreover, future research could shed light on how individuals interpret step count. For example, a possible reason why the inflated step count condition failed to induce more adequate activity mindsets (compared with accurate step count) is that people may be less sensitive to changes in step count beyond a certain threshold. Participants reached approximately 7000 steps per day on average, which translates into a deflated step count of 4200 and an inflated step count of 9800. Participants may have perceived 7000 and 9800 steps as qualitatively similar (ie, in the high thousands but below the common 10,000-step target), whereas 4200 may have seemed qualitatively different (ie, <5000 steps and, thus, relatively low). This is consistent with previous findings that mindsets are a subjective interpretation of reality, which can be influenced by factors such as social comparison, guidelines, and targets [24,25].

Finally, future research could examine the role of mindsets in predicting wearable tracker adoption, abandonment, and user engagement, which are major obstacles to trackers’ effectiveness as well as their systematic evaluation [11,64]. To date, wearable devices are not widely used by individuals who stand to benefit the most from monitoring and improvement (including older individuals and those with poor health or chronic conditions) [64]. It may be that these individuals have inadequate activity mindsets and are afraid of being constantly reminded of their perceived unhealthy lifestyle or that they quickly become discouraged by feedback that their activity levels are inadequate. Mindset interventions may help buffer individuals from these negative effects and promote adoption and sustained engagement in diverse populations.

### Conclusions

Physical activity is a critical determinant of health and well-being. This research suggests that it is not only our actual physical activity behavior that matters but also our mindsets about the adequacy and health consequences of our physical activity. Moreover, it shows that wearable fitness trackers can shape these mindsets. These insights may be used to support the design of wearable trackers and other health technologies that more effectively boost users’ health and well-being. In addition, health psychology research and public health policy may design more successful public health interventions by more deliberately––and more effectively––harnessing the power of mindsets.

## Appendix

Multimedia Appendix 1 Supplemental methods, materials, and results.

Multimedia Appendix 2 CONSORT-eHEALTH checklist (V 1.6.1).

## Abbreviations

AAM: activity adequacy mindset
BCT: behavior change technique
DBP: diastolic blood pressure
HR: heart rate
MAP: mean arterial pressure
PROMIS: Patient-Reported Outcomes Measurement Information System
SBP: systolic blood pressure
