# Supplementary material for: Effects of Wearable Fitness Trackers and Activity Adequacy Mindsets on Affect, Behavior, and Health: Longitudinal Randomized Controlled Trial
Source: J Med Internet Res. 2023 Jan 25;25:e40529. doi: 10.2196/40529 (PMC9909519; doi:10.2196/40529)
Supplement: Multimedia Appendix 1 [file jmir_v25i1e40529_app1.doc]

**Supplemental Materials for**

**Effects of Wearable Fitness Trackers and Activity Adequacy Mindsets on Affect, Behavior and Health: A Longitudinal Randomized Controlled Trial**

Octavia H. Zahrt^1^, Kristopher E. Evans^2^, Elizabeth L. Murnane^3^, Erik W. Santoro^2^, Michael Baiocchi^4^,

James A. Landay^3^, Scott L. Delp^5^, and Alia J. Crum^2^

^1^ Department of Organizational Behavior, Stanford Graduate School of Business, Stanford University

^2^ Department of Psychology, Stanford University; ^3^ Department of Computer Science, Stanford University

^4^ Department of Epidemiology and Population Health, Stanford University

^5^ Department of Mechanical Engineering, Department of Bioengineering, Stanford University

﻿Correspondence concerning this article should be addressed to Alia Crum, ﻿Department of Psychology, Stanford University, 450 Serra Mall, Stanford, CA 94305. E-mail: crum@stanford.edu.

**Contents:**

Supplemental Methods and Materials

Supplemental Results

Appendix

# Supplemental Methods & Materials [[1]](#footnote-2)

## Participants and Procedure

Participants were a diverse sample of Northern California community-dwelling adults recruited via flyers and online platforms (i.e., Craigslist, Nextdoor) between 09/2017 and 09/2019. The posting advertised an opportunity to participate in a paid research study to develop more effective fitness trackers. Individuals interested in participating were asked to take a short pre-screen survey to ensure eligibility (see Appendix). Qualified participants were invited to join the study if they met the following criteria: walking as the primary source of physical activity in the prior six months; health status allows engagement in physical activity according to the Physical Activity Readiness Questionnaire [51]; not pregnant (as natural changes in weight and body composition during pregnancy would invalidate results); possession of an iPhone 5 S or newer (to allow connecting an Apple Watch); and limited exposure to activity tracking technology or apps (to ensure participants were naïve to their daily step count). Our target sample size was 160, but we overrecruited due to projected attrition. A total of 207 volunteers were randomized to one of four conditions (accurate, deflated, inflated and meta-mindset intervention).

During the in-lab onboarding session, participants were briefed with the cover story; received instructions for study participations, an Apple Watch and various materials (see Appendix for onboarding scripts and materials); and completed psychological and physiological assessments.

Throughout the following 5 weeks, participants’ step count was tracked by Apple Watch. Additionally, they completed weekly surveys and daily check-ins by following Qualtrics survey links sent via text message to their phones[[2]](#footnote-3) at 8 PM each night. The weekly surveys assessed affective and behavioral process measures, whereas the daily check-ins asked participants to enter their current step count (to ensure that they were aware of their step count) and asked about technological issues and watch wear times.

Participants’ study adherence was monitored on a daily basis. In particular, researchers used a dashboard developed for this study to monitor Apple Watch-tracked step count and heart rate data. They communicated with participants via email, text message and phone when no data updates were registered to the cloud database for three hours during daytime to remind participants to wear the watch or troubleshoot any technical issues. Additionally, survey responses were tracked and encouraged when necessary.

During the offboarding session, participants were debriefed and asked about compliance with study instructions. They were paid $175 for satisfactory participation (deductions were made in case of significant non-compliance, Apple Watch loss or damage).

Suspicions regarding the experimental manipulations and study purpose were probed with up to ten progressively specific questions (see Appendix). The researcher then assigned a rating of how suspicious a participant was about study purpose/ deception, and of whether a participant seemed to be lying regarding adhering to the protocol/ not looking at other trackers, etc. (1 – not at all; 2 – slightly; 3 – somewhat; 4 – very; 5 – extremely; please explain).

Forty-five participants were excluded from data analysis due to early drop-out (n = 9), non-compliance with instructions (i.e., using other activity-tracking applications or comparing step count with friends; n = 9), survey completion below 80 % (n = 8), failure to complete the meta-mindset intervention (n = 7; some due to a technical issue), offboarding session completed more than three days late (n = 4), significant suspicion about study manipulations (i.e., correctly guessed that step count was inflated or deflated, with suspicion rating above 3; n = 4), Apple Watch activity recorded on fewer than 80 % of study days (n = 2), or pregnancy (n = 2). A total of 162 participants completed the study satisfactorily. See Figure S1 for a Consort flow diagram and Table S1 for participant baseline characteristics.

The study was approved by the Stanford University IRB (protocol ID: 36098). Informed consent was required for study participation (see Appendix).

## Randomization

Participants were assigned to experimental condition via criteria-based randomization (CBR), a novel procedure that helps minimize imbalances in pre-manipulation covariate distributions across experimental groups in order to increase precision and statistical power [52, 53]. ﻿CBR helps create intervention assignments that the analyst believes will generate data useful for obtaining defensible causal estimates by creating experimental groups which have (approximately) equivalent pre-intervention covariate distributions (see original papers for details). To do so, the analyst specifies a set of pre-intervention covariate measurements on which they would like to achieve balance across groups, along with relative weights determining which covariates are more or less important to balance. This research used all available pre-intervention measures in the algorithm (see R code for exact procedure and weighting).

Random assignment was conducted in between onboarding and day 8 of study participation by an experimenter who did not interact with participants to ensure double blindness.

**Figure S1.** CONSORT flow diagram showing the flow of progress through the study’s different phases.

**Table S1.** Participants’ socio-demographic characteristics (total sample and by experimental condition). Count and proportion in percent are displayed unless otherwise noted.

|  | Total Sample (N=162) | Accurate (N=41) | Deflated (N=40) | Inflated (N=40) | Meta (N=41) |
| --- | --- | --- | --- | --- | --- |
| Age: median (range) | 40 (19-76) | 42 (20-76) | 41.5 (20-70) | 39 (19-69) | 39 (20-67) |
| Gender |  |  |  |  |  |
| female | 118 (73%) | 31 (76%) | 30 (75%) | 26 (65%) | 31 (76%) |
| male | 43 (27%) | 10 (24%) | 10 (25%) | 13 (32%) | 10 (24%) |
| Other | 1 (1%) | 0 (0%) | 0 (0%) | 1 (2%) | 0 (0%) |
| Race/ Ethnicity |  |  |  |  |  |
| Asian (American) | 40 (25%) | 12 (29%) | 8 (20%) | 10 (25%) | 10 (24%) |
| Black/ African American | 13 (8%) | 2 (5%) | 2 (5%) | 3 (8%) | 6 (15%) |
| Hispanic | 18 (11%) | 4 (10%) | 2 (5%) | 7 (18%) | 5 (12%) |
| White/ European American | 70 (43%) | 17 (41%) | 22 (55%) | 14 (35%) | 17 (41%) |
| Mixed | 13 (8%) | 3 (7%) | 4 (10%) | 4 (10%) | 2 (5%) |
| Other | 8 (5%) | 3 (7%) | 2 (5%) | 2 (5%) | 1 (2%) |
| Education |  |  |  |  |  |
| High School Degree or less | 5 (3%) | 1 (2%) | 1 (2%) | 1 (2%) | 2 (5%) |
| Some College | 33 (20%) | 9 (22%) | 9 (22%) | 6 (15%) | 9 (22%) |
| 2-Year Degree | 11 (7%) | 1 (2%) | 3 (8%) | 5 (12%) | 2 (5%) |
| 4-Year Degree | 71 (44%) | 20 (49%) | 17 (42%) | 17 (42%) | 17 (41%) |
| Masters/ Professional Degree | 32 (20%) | 5 (12%) | 8 (20%) | 9 (22%) | 10 (24%) |
| Doctorate Degree | 10 (6%) | 5 (12%) | 2 (5%) | 2 (5%) | 1 (2%) |
| Employment |  |  |  |  |  |
| Employed | 75 (46%) | 17 (41%) | 17 (42%) | 19 (48%) | 22 (54%) |
| Self-Employed | 44 (27%) | 13 (32%) | 13 (32%) | 9 (22%) | 9 (22%) |
| Homemaker | 7 (4%) | 2 (5%) | 2 (5%) | 2 (5%) | 1 (2%) |
| Student | 1 (1%) | 0 (0%) | 0 (0%) | 1 (2%) | 0 (0%) |
| Unemployed | 25 (15%) | 5 (12%) | 6 (15%) | 7 (18%) | 7 (17%) |
| Retired | 7 (4%) | 4 (10%) | 0 (0%) | 2 (5%) | 1 (2%) |
| Unable to Work | 3 (2%) | 0 (0%) | 2 (5%) | 0 (0%) | 1 (2%) |
| Marital Status |  |  |  |  |  |
| Married | 56 (35%) | 17 (41%) | 10 (25%) | 16 (40%) | 13 (32%) |
| Unmarried Couple | 14 (9%) | 1 (2%) | 5 (12%) | 4 (10%) | 4 (10%) |
| Single | 73 (45%) | 20 (49%) | 20 (50%) | 15 (38%) | 18 (44%) |
| Divorced/ Separated/ Widowed | 19 (12%) | 3 (7%) | 5 (12%) | 5 (12%) | 6 (15%) |
| Caretaker |  |  |  |  |  |
| yes | 54 (33%) | 13 (32%) | 19 (48%) | 13 (32%) | 9 (22%) |
| no | 108 (67%) | 28 (68%) | 21 (52%) | 27 (68%) | 32 (78%) |

## Measures

Measures were collected during the onboarding and offboarding sessions using iPads for the survey and a digital health monitors for physiological assessments. In addition, participants completed weekly online surveys, which took approximately 10-15 minutes to complete and measured a subset of the onboarding survey measures (i.e., the processes hypothesized to explain the effects of activity adequacy mindset on health and wellbeing).

### Activity Adequacy Mindset (AAM; Manipulation Check)

Measured in weekly surveys. Participants’ mindset that their physical activity was adequate and healthy or inadequate and unhealthy was assessed using a shortened 5-item version of the Activity Adequacy Mindset Scale [24]. Items include “My current level of physical activity is unhealthy” (7-point scale: 1 = *Strongly disagree* – 7 = *Strongly agree*); “My current level of physical activity is helping me achieve or maintain a healthy body weight” (7-point scale: 1 = *Strongly disagree* – 7 = *Strongly agree*); “How beneficial is your current level of physical activity for your health?” (5-point scale: 1 = *Not at all beneficial* – 5 = *Extremely beneficial*); “How much is your current level of physical (in-)activity strengthening or weakening your muscles?” (7-point scale: 1 = *Strengthening very much* – 7 = *Weakening very much*); “How much does your current level of physical (in-)activity increase or decrease your risk of disease?” (7-point scale: 1 = *Increases my risk very much* – 7 = *Decreases my risk very much*). After reverse-scoring and scaling relevant items, a composite average score ranging from 1 to 7 was calculated (Cronbach’s ﻿α ≥ 0.85). A higher score on the activity adequacy mindset measure indicates greater agreement with the mindset that an individuals’ current level of physical activity is adequate and healthy.

### Health Outcomes

*Physiological health* was assessed in the lab using digital health monitors. Body weight (in lbs) was measured in lab using Tanita BC-544 Body Composition Monitor. An Omron blood pressure monitor measured resting systolic blood pressure (SBP) and diastolic blood pressure (DBP). They were then combined to calculate mean arterial pressure (MAP, i.e., an individual’s average blood pressure during a single cardiac cycle) for statistical analysis, according to the formula: *MAP = (SBP + 2 * DBP) / 3.* Resting heart rate was measured via SantaMedical SM-165 finger sensor. Maximal aerobic capacity (i.e., VO_2_ max) was measured using the Canadian Home Fitness Test [54]. Participants stepped up and down an aerobics step at an age- and sex-specific rhythm for three minutes. Immediately following this activity, heart rate was measured using the SantaMedical SM-165 finger sensor. Participants completed up to two additional three-minute sessions with increasing stepping pace if heart rate was below age- and sex-specific cutoff level. Based on the final post-activity heart rate, age, body weight, and energy requirement estimated depending on sex and stepping stage reached, aerobic capacity was calculated according to the formula [54]: *VO_2_ max = 42.5 + 16.6 * (energy requirement) - 0.12 * (body weight) - 0.12 * (final heart rate) - 0.24 * (age).*

### Affective Processes

The Affect Valuation Index [59] actual affect subscale was used to measure how often during the past seven/ thirty days participants felt 25 affective states (5-point scale: 1 = *Never* – 5 = *All the time*). Two composite scores were created for analysis, one by averaging all items measuring positive affective states (happy, content, satisfied, enthusiastic, excited, euphoric, elated, calm, relaxed, peaceful, serene; Cronbach’s ﻿α ≥ 0.87), and another by averaging all items measuring negative affective states (unhappy, sad, fearful, nervous, hostile, dull, sleepy, sluggish; Cronbach’s ﻿α ≥ 0.79).

### Behavioral Processes

Engagement in physical activities other than walking was measured via a self-report measure developed for this study (adapted from measures used in the National Health and Nutrition Examination Survey, NHANES). The measure was presented as a general measure of various activities that participants might have engaged in during the past seven/ thirty days. It included traditional exercise activities (e.g., “Sports (e.g., football, basketball, tennis)”; “Swimming or water sports”), physical activities that people might not consider exercise (e.g., “Housekeeping work”; “Lifting or carrying children, shopping bags, or other heavy loads”), and non-physical activities as filler items (e.g., “Playing board games or video games”). For each of the 25 activities, participants indicated having done; they also reported frequency (number of times during the past 7 days), average duration per occasion (in hours and minutes), and intensity of physical or mental effort (4-point scale: 0 = *No physical effort*, 1 = *Light physical effort*, 2 = *Moderate physical effort*, 3 = *Vigorous physical effort*; or equivalent scale for mental effort). Only the physical activities were used for analysis. Among physical activities, those redundant with the Apple Watch-tracked step count were excluded (i.e., walking; hiking at no or light physical effort; jogging/ running at no or light physical effort; stair climbing at no or light physical effort). Next, the metabolic equivalent of task ratings quantifying energy expenditure associated with an activity [55] was assigned to each physical activity at each intensity level. Finally, average total energy expenditure in kilocalories per day was computed as a measure of physical activity used in analyses.

*Other health-relevant behaviors* were also measured via self-report. Dietary choices during the past seven/ thirty days were assessed via a total of four items, measuring the consumption of high-fat foods (“fatty meats, fatty cheese, sour cream, butter, French fries, pizza, etc.”); sugary foods or drinks (“sodas, candy, donuts, cookies, pastries, ice cream or other deserts”); healthy produce (“servings of fruits, vegetables, or salads”; all assessed on 8-point scales: 0 = *none at all*, 1 = *1 per week*, 2 = *2-4 per week*, 3 = *5-6 per week*, 4 = *1 per day*, 5 = *2-3 per day*, 6 = *4-5 per day*, 7 = *6+ per day*); as well as overeating (“During the past 7 days/ 30 days, how often did you eat too much?”; 5-point scale: 0 = *not at all*, 1 = *1-2 times a week*, 2 = *3-4 times a week*, 3 = *5-6 times a week*, 4 = *Every day*). Smoking behavior and alcohol consumption were assessed using two items each (“During the past 7/ 30 days, on how many days did you smoke cigarettes?” 3-point scale: 0 = *No days,* 1 = *Some days,* 2 = *Every day or most days*; “During the past 7 days, how many cigarettes did you smoke on a typical day that you smoked?” 5-point scale: 1 = *5 cigarettes or less*, 2 = *6-10*, 3 = *11-20*, 4 = *21-30*, 5 = *31 or more*; “During the past 7/ 30 days, how often did you usually have any kind of drink containing alcohol?” 6-point scale: 0 = *not at all*, 1 = *less than once* *a week*, 2 = *1-2 times a week*, 3 = *3-4 times a week*, 4 = *5-6 times a week*, 5 = *Every day*; “During the past 7/ 30 days, how many alcoholic drinks did you have on a typical day when you drank alcohol?” 7-point scale: 1 = *1 drink*, 2 = *2 drinks*, 3 = *3-4 drinks*, 4 = *5-6 drinks*, 5 = *7-10 drinks*, 6 = *11-15 drinks*, 7 = *16 drinks or more*). Items were combined through multiplication into two summary measures to indicate overall smoking and alcohol consumption, respectively.

## Analytical approach[[3]](#footnote-4)

### Preliminary Analysis

For health and wellbeing outcomes (measured during onboarding and offboarding), linear regression models were fitted to examine differences by condition in baseline levels:

*health.measure _i j_  = γ_0_ + γ_1_  condition _i_ + ϵ _i j_*

For AAM, affective and behavioral processes (measured multiple times during baseline period), multilevel models were fitted to examine baseline differences:

Level I Model (within-subjects):
*process.measure _i j_ = α _i_ + ϵ _i j_*

- α _i_ : baseline level for the i’th participant

Level II Model (between subjects):
*α_i_ = γ_0_ + γ_1_ condition _i_ + u _i_*

- baseline level depends on experimental condition
- u _i_ : random intercept for the i’th participant

Combined Model (obtained by plugging Level II into Level I):
*process.measure _i j_ = γ_0_ + γ_1_ condition _i_ + ϵ _i j_ + u _i_*

- a significant fixed effect for condition indicates that experimental condition significantly predicts baseline level

If baseline differences were detected, the main analysis included a fixed effect for condition at baseline to accurately model changes from baseline to treatment periods.

### Main Analysis

Multilevel longitudinal models were used to predict changes in health and wellbeing outcomes, AAM, affective and behavioral processes from baseline to treatment period. All models (except those predicting physical activity changes) adjusted for two covariates––step count and other self-reported physical activities––to test whether mindset manipulations affected outcomes independently of actual physical activity:

Level I Model (within subjects):
*measure _i j_  = α_0 i_ + α_1 i_ treat _i j_ + α_2 i_ stepcount _i j_  + α_3 i_ otherPA _i j_  + ϵ _i j_*

- α_0 i_ : intercept, baseline level for the i’th participant
- α_1 i_ : slope, change from baseline to treatment period for the i’th participant (adding α_0 i_ and α_1 i_ gives treatment period intercept)
- treat _i j_ : binary indicator for baseline (0) vs. treatment (1) period
- α_2 i_ : slope, effect of step count
- α_3 i_ : slope, effect of other self-reported physical activities
- ϵ _i j_ : residual for the i’th participant on the j’th day

Level II Model (between subjects):
*α_0 i_ = γ_00_ [ + γ_01_ condition _i_ ] + u_0 i_*

- baseline level is just the population estimate γ_00_ plus random intercept u_0i_; in case baseline differences were detected in preliminary analysis, γ_01_ estimates the effect of condition

*α_1 i_ = γ_10_ + γ_11_ condition _i_ [ + u_1 i_ ]*

- change from baseline to treatment period depends on experimental condition
- u_1 i_ : random slope for the i’th participant, included only in models predicting AAM, affective and behavioral processes due to degrees of freedom

*α_2 i_ = γ_20_*

- effect of step count is just the population estimate γ_20_

*α_3 i_ = γ_30_*

- effect of other self-reported physical activities is just the population estimate γ_30_

Combined Model (obtained by plugging Level II into Level I):

*measure _i j_  =  γ_00_ [ + γ_01_ condition _i_ ] +*

*γ_10_ treat _i j_ + γ_11_ treat _i j_ condition _i_ +*

*γ_20_ stepcount _i j_ + γ_30_ otherPA _i j_ +*

*ϵ _i j_ + u_0 i_ [ + u_1 i_ ]*

- a significant treat X condition interaction indicates that experimental condition significantly changes measure

As described in the main report, AAM, affective and behavioral processes are theorized as mechanisms explaining changes in health. Thus, only their weekly (not onboarding/ offboarding) measurements were used in Model 1 (i.e., the model examining changes within the accurate step count condition from baseline to treatment, and compared changes in the accurate condition (reference group) with changes in the deflated and inflated conditions). However, the meta-mindset intervention was delivered in the first weekly survey before assessment of AAM, affect, diet, smoking, and alcohol. Thus, Model 2 (i.e., the model comparing changes from baseline to treatment in the accurate condition (reference group) with the meta-mindset intervention condition) included onboarding (the only baseline measurement), weekly, and offboarding for these variables. See Table S2.

***Table S2.*** *Measurements used for the two models*

|  | Model 1: Inflated deflated vs. accurate | Model 2: Meta vs. accurate |
| --- | --- | --- |
| AAM | Weekly (week 1 = baseline) | Onboarding, offboarding, weekly (onboarding = baseline) |
| Affect, behavior | Weekly (week 1 = baseline) | Onboarding, offboarding, weekly (onboarding = baseline) |
| Health | Onboarding, offboarding (onboarding = baseline) | |
| Self-report physical activity | Weekly (week 1 = baseline) | |
| Step count | Daily (days 1-7 = baseline) | |

# Supplemental Results

## Effects of Accurate and Manipulated Step Count Feedback

### Activity Adequacy Mindset

Multilevel longitudinal model; without physical activity covariates

|  | Estimate | SE | t-value | p-value |
| --- | --- | --- | --- | --- |
| (Intercept) | 3.194 | 0.162 | 19.773 | 0.000 |
| Deflated Condition (vs. Accurate) | 0.533 | 0.244 | 2.186 | 0.031 |
| Inflated Condition (vs. Accurate) | 0.354 | 0.269 | 1.316 | 0.191 |
| Treatment Period (vs. Baseline) | 0.291 | 0.103 | 2.836 | 0.005 |
| Deflated Condition X Treatment Period | -0.447 | 0.161 | -2.767 | 0.006 |
| Inflated Condition X Treatment Period | -0.140 | 0.163 | -0.862 | 0.389 |

Multilevel longitudinal model; with physical activity covariates

|  | Estimate | SE | t-value | p-value |
| --- | --- | --- | --- | --- |
| (Intercept) | 2.191 | 0.210 | 10.456 | 0.000 |
| Deflated Condition (vs. Accurate) | 0.402 | 0.226 | 1.775 | 0.078 |
| Inflated Condition (vs. Accurate) | 0.213 | 0.251 | 0.849 | 0.398 |
| Treatment Period (vs. Baseline) | 0.265 | 0.099 | 2.671 | 0.008 |
| Step Count | 0.000 | 0.000 | 6.715 | 0.000 |
| Other Physical Activities | 0.029 | 0.012 | 2.526 | 0.012 |
| Deflated Condition X Treatment Period | -0.397 | 0.160 | -2.475 | 0.014 |
| Inflated Condition X Treatment Period | -0.079 | 0.157 | -0.505 | 0.614 |

### Health Outcomes

#### Functional Health.

Multilevel longitudinal model; without physical activity covariates

|  | Estimate | SE | t-value | p-value |
| --- | --- | --- | --- | --- |
| (Intercept) | 4.481 | 0.052 | 85.413 | 0.000 |
| Treatment Period (vs. Baseline) | -0.113 | 0.049 | -2.326 | 0.021 |
| Deflated Condition X Treatment Period | -0.030 | 0.087 | -0.347 | 0.729 |
| Inflated Condition X Treatment Period | 0.050 | 0.099 | 0.504 | 0.615 |

Multilevel longitudinal model; with physical activity covariates

|  | Estimate | SE | t-value | p-value |
| --- | --- | --- | --- | --- |
| (Intercept) | 4.462 | 0.061 | 73.679 | 0.000 |
| Treatment Period (vs. Baseline) | -0.117 | 0.049 | -2.387 | 0.018 |
| Step Count | 0.098 | 0.037 | 2.618 | 0.009 |
| Other Physical Activities | 0.004 | 0.006 | 0.693 | 0.489 |
| Deflated Condition X Treatment Period | -0.022 | 0.086 | -0.257 | 0.797 |
| Inflated Condition X Treatment Period | 0.059 | 0.097 | 0.606 | 0.545 |

#### Mental Health.

Multilevel longitudinal model; without physical activity covariates

|  | Estimate | SE | t-value | p-value |
| --- | --- | --- | --- | --- |
| (Intercept) | 3.741 | 0.063 | 59.160 | 0.000 |
| Treatment Period (vs. Baseline) | 0.154 | 0.070 | 2.210 | 0.029 |
| Deflated Condition X Treatment Period | -0.212 | 0.090 | -2.362 | 0.019 |
| Inflated Condition X Treatment Period | -0.041 | 0.099 | -0.409 | 0.683 |

Multilevel longitudinal model; with physical activity covariates

|  | Estimate | SE | t-value | p-value |
| --- | --- | --- | --- | --- |
| (Intercept) | 3.700 | 0.071 | 52.475 | 0.000 |
| Treatment Period (vs. Baseline) | 0.151 | 0.071 | 2.139 | 0.034 |
| Step Count | 0.045 | 0.044 | 1.045 | 0.297 |
| Other Physical Activities | 0.008 | 0.006 | 1.310 | 0.192 |
| Deflated Condition X Treatment Period | -0.202 | 0.091 | -2.219 | 0.028 |
| Inflated Condition X Treatment Period | -0.033 | 0.098 | -0.338 | 0.736 |

#### Self-Esteem.

Multilevel longitudinal model; without physical activity covariates

|  | Estimate | SE | t-value | p-value |
| --- | --- | --- | --- | --- |
| (Intercept) | 3.868 | 0.071 | 54.245 | 0.000 |
| Treatment Period (vs. Baseline) | 0.089 | 0.058 | 1.526 | 0.129 |
| Deflated Condition X Treatment Period | -0.235 | 0.113 | -2.072 | 0.040 |
| Inflated Condition X Treatment Period | -0.109 | 0.112 | -0.975 | 0.331 |

Multilevel longitudinal model; with physical activity covariates

|  | Estimate | SE | t-value | p-value |
| --- | --- | --- | --- | --- |
| (Intercept) | 3.771 | 0.080 | 47.065 | 0.000 |
| Treatment Period (vs. Baseline) | 0.114 | 0.059 | 1.917 | 0.057 |
| Step Count | -0.077 | 0.054 | -1.423 | 0.156 |
| Other Physical Activities | 0.018 | 0.008 | 2.407 | 0.017 |
| Deflated Condition X Treatment Period | -0.241 | 0.111 | -2.174 | 0.031 |
| Inflated Condition X Treatment Period | -0.140 | 0.109 | -1.279 | 0.203 |

#### Bodyweight.

Multilevel longitudinal model; without physical activity covariates

|  | Estimate | SE | t-value | p-value |
| --- | --- | --- | --- | --- |
| (Intercept) | 155.215 | 6.310 | 24.599 | 0.000 |
| Deflated Condition (vs. Accurate) | 13.340 | 8.821 | 1.512 | 0.133 |
| Inflated Condition (vs. Accurate) | 28.855 | 11.413 | 2.528 | 0.013 |
| Treatment Period (vs. Baseline) | -0.044 | 0.421 | -0.104 | 0.917 |
| Deflated Condition X Treatment Period | -0.271 | 0.620 | -0.438 | 0.663 |
| Inflated Condition X Treatment Period | -1.268 | 1.112 | -1.141 | 0.256 |

Multilevel longitudinal model; with physical activity covariates

|  | Estimate | SE | t-value | p-value |
| --- | --- | --- | --- | --- |
| (Intercept) | 155.468 | 6.319 | 24.602 | 0.000 |
| Deflated Condition (vs. Accurate) | 13.468 | 8.834 | 1.525 | 0.130 |
| Inflated Condition (vs. Accurate) | 28.969 | 11.409 | 2.539 | 0.012 |
| Treatment Period (vs. Baseline) | -0.121 | 0.437 | -0.276 | 0.783 |
| Step Count | -0.501 | 0.593 | -0.845 | 0.400 |
| Other Physical Activities | -0.058 | 0.088 | -0.662 | 0.509 |
| Deflated Condition X Treatment Period | -0.268 | 0.625 | -0.430 | 0.668 |
| Inflated Condition X Treatment Period | -1.246 | 1.166 | -1.068 | 0.288 |

#### VO2 Max.

Multilevel longitudinal model; without physical activity covariates

|  | Estimate | SE | t-value | p-value |
| --- | --- | --- | --- | --- |
| (Intercept) | 31.394 | 0.706 | 44.462 | 0.000 |
| Treatment Period (vs. Baseline) | 0.565 | 0.311 | 1.818 | 0.072 |
| Deflated Condition X Treatment Period | -0.309 | 0.443 | -0.698 | 0.487 |
| Inflated Condition X Treatment Period | -0.508 | 0.509 | -0.997 | 0.321 |

Multilevel longitudinal model; with physical activity covariates

|  | Estimate | SE | t-value | p-value |
| --- | --- | --- | --- | --- |
| (Intercept) | 31.554 | 0.726 | 43.451 | 0.000 |
| Treatment Period (vs. Baseline) | 0.564 | 0.315 | 1.787 | 0.077 |
| Step Count | 0.172 | 0.314 | 0.548 | 0.585 |
| Other Physical Activities | -0.033 | 0.038 | -0.878 | 0.381 |
| Deflated Condition X Treatment Period | -0.354 | 0.452 | -0.783 | 0.435 |
| Inflated Condition X Treatment Period | -0.487 | 0.503 | -0.967 | 0.335 |

#### Mean Arterial Blood Pressure.

Multilevel longitudinal model; without physical activity covariates

|  | Estimate | SE | t-value | p-value |
| --- | --- | --- | --- | --- |
| (Intercept) | 92.749 | 1.106 | 83.888 | 0.000 |
| Treatment Period (vs. Baseline) | -2.337 | 1.515 | -1.543 | 0.125 |
| Deflated Condition X Treatment Period | 3.698 | 1.792 | 2.064 | 0.041 |
| Inflated Condition X Treatment Period | 3.605 | 1.828 | 1.971 | 0.050 |

Multilevel longitudinal model; with physical activity covariates

|  | Estimate | SE | t-value | p-value |
| --- | --- | --- | --- | --- |
| (Intercept) | 92.674 | 1.214 | 76.367 | 0.000 |
| Treatment Period (vs. Baseline) | -2.267 | 1.548 | -1.464 | 0.145 |
| Step Count | 0.029 | 0.703 | 0.041 | 0.968 |
| Other Physical Activities | 0.010 | 0.106 | 0.091 | 0.928 |
| Deflated Condition X Treatment Period | 3.641 | 1.825 | 1.995 | 0.048 |
| Inflated Condition X Treatment Period | 3.546 | 1.869 | 1.897 | 0.060 |

#### Resting Heart Rate.

Multilevel longitudinal model; without physical activity covariates

|  | Estimate | SE | t-value | p-value |
| --- | --- | --- | --- | --- |
| (Intercept) | 71.293 | 0.930 | 76.631 | 0.000 |
| Treatment Period (vs. Baseline) | 0.594 | 1.306 | 0.455 | 0.650 |
| Deflated Condition X Treatment Period | 3.482 | 1.842 | 1.890 | 0.061 |
| Inflated Condition X Treatment Period | 2.946 | 1.757 | 1.677 | 0.096 |

Multilevel longitudinal model; with physical activity covariates

|  | Estimate | SE | t-value | p-value |
| --- | --- | --- | --- | --- |
| (Intercept) | 71.793 | 1.054 | 68.093 | 0.000 |
| Treatment Period (vs. Baseline) | 0.413 | 1.233 | 0.335 | 0.738 |
| Step Count | -2.348 | 0.681 | -3.450 | 0.001 |
| Other Physical Activities | -0.077 | 0.110 | -0.697 | 0.486 |
| Deflated Condition X Treatment Period | 3.542 | 1.786 | 1.983 | 0.049 |
| Inflated Condition X Treatment Period | 2.949 | 1.641 | 1.797 | 0.074 |

### Affective Processes

#### Negative Affect.

Multilevel longitudinal model; without physical activity covariates

|  | Estimate | SE | t-value | p-value |
| --- | --- | --- | --- | --- |
| (Intercept) | 2.074 | 0.048 | 43.506 | 0.000 |
| Treatment Period (vs. Baseline) | -0.072 | 0.046 | -1.562 | 0.119 |
| Deflated Condition X Treatment Period | 0.118 | 0.066 | 1.791 | 0.074 |
| Inflated Condition X Treatment Period | -0.004 | 0.067 | -0.058 | 0.954 |

Multilevel longitudinal model; with physical activity covariates

|  | Estimate | SE | t-value | p-value |
| --- | --- | --- | --- | --- |
| (Intercept) | 2.195 | 0.076 | 28.757 | 0.000 |
| Treatment Period (vs. Baseline) | -0.068 | 0.046 | -1.476 | 0.142 |
| Step Count | 0.000 | 0.000 | -2.082 | 0.038 |
| Other Physical Activities | 0.002 | 0.003 | 0.595 | 0.552 |
| Deflated Condition X Treatment Period | 0.118 | 0.065 | 1.814 | 0.072 |
| Inflated Condition X Treatment Period | -0.012 | 0.067 | -0.179 | 0.858 |

#### Positive Affect.

Multilevel longitudinal model; without physical activity covariates

|  | Estimate | SE | t-value | p-value |
| --- | --- | --- | --- | --- |
| (Intercept) | 2.543 | 0.094 | 27.151 | 0.000 |
| Deflated Condition (vs. Accurate) | 0.062 | 0.136 | 0.454 | 0.650 |
| Inflated Condition (vs. Accurate) | 0.335 | 0.149 | 2.253 | 0.025 |
| Treatment Period (vs. Baseline) | -0.039 | 0.065 | -0.593 | 0.554 |
| Deflated Condition X Treatment Period | -0.011 | 0.096 | -0.119 | 0.905 |
| Inflated Condition X Treatment Period | -0.098 | 0.088 | -1.106 | 0.269 |

Multilevel longitudinal model; with physical activity covariates

|  | Estimate | SE | t-value | p-value |
| --- | --- | --- | --- | --- |
| (Intercept) | 2.455 | 0.119 | 20.607 | 0.000 |
| Deflated Condition (vs. Accurate) | 0.027 | 0.137 | 0.198 | 0.844 |
| Inflated Condition (vs. Accurate) | 0.318 | 0.151 | 2.106 | 0.037 |
| Treatment Period (vs. Baseline) | -0.037 | 0.065 | -0.570 | 0.570 |
| Step Count | 0.000 | 0.000 | 0.583 | 0.560 |
| Other Physical Activities | 0.011 | 0.004 | 2.798 | 0.005 |
| Deflated Condition X Treatment Period | 0.000 | 0.094 | 0.005 | 0.996 |
| Inflated Condition X Treatment Period | -0.097 | 0.089 | -1.096 | 0.276 |

### Behavioral Processes

#### Step Count.

Multilevel longitudinal model; without physical activity covariates

|  | Estimate | SE | t-value | p-value |
| --- | --- | --- | --- | --- |
| (Intercept) | 7100.089 | 237.922 | 29.842 | 0.000 |
| Treatment Period (vs. Baseline) | 124.970 | 254.654 | 0.491 | 0.624 |
| Deflated Condition X Treatment Period | -149.143 | 346.983 | -0.430 | 0.668 |
| Inflated Condition X Treatment Period | -292.009 | 349.394 | -0.836 | 0.405 |

#### Other Physical Activities.

Multilevel longitudinal model; without physical activity covariates

|  | Estimate | SE | t-value | p-value |
| --- | --- | --- | --- | --- |
| (Intercept) | 3.819 | 0.547 | 6.978 | 0.000 |
| Deflated Condition (vs. Accurate) | 2.933 | 1.481 | 1.98 | 0.049 |
| Inflated Condition (vs. Accurate) | 1.027 | 0.989 | 1.038 | 0.300 |
| Treatment Period (vs. Baseline) | -0.375 | 0.508 | -0.738 | 0.461 |
| Deflated Condition X Treatment Period | -0.914 | 0.961 | -0.951 | 0.342 |
| Inflated Condition X Treatment Period | 0.316 | 1.000 | 0.316 | 0.752 |

#### Overeating.

Multilevel longitudinal model; without physical activity covariates

|  | Estimate | SE | t-value | p-value |
| --- | --- | --- | --- | --- |
| (Intercept) | 0.857 | 0.119 | 7.223 | 0.000 |
| Deflated Condition (vs. Accurate) | -0.032 | 0.166 | -0.192 | 0.848 |
| Inflated Condition (vs. Accurate) | 0.543 | 0.209 | 2.594 | 0.011 |
| Treatment Period (vs. Baseline) | 0.053 | 0.084 | 0.624 | 0.534 |
| Deflated Condition X Treatment Period | 0.061 | 0.115 | 0.534 | 0.594 |
| Inflated Condition X Treatment Period | -0.249 | 0.178 | -1.402 | 0.163 |

Multilevel longitudinal model; with physical activity covariates

|  | Estimate | SE | t-value | p-value |
| --- | --- | --- | --- | --- |
| (Intercept) | 0.970 | 0.165 | 5.863 | 0.000 |
| Deflated Condition (vs. Accurate) | -0.013 | 0.166 | -0.079 | 0.937 |
| Inflated Condition (vs. Accurate) | 0.560 | 0.209 | 2.673 | 0.009 |
| Treatment Period (vs. Baseline) | 0.055 | 0.084 | 0.652 | 0.516 |
| Step Count | 0.000 | 0.000 | -0.790 | 0.430 |
| Other Physical Activities | -0.005 | 0.006 | -0.810 | 0.418 |
| Deflated Condition X Treatment Period | 0.055 | 0.114 | 0.480 | 0.632 |
| Inflated Condition X Treatment Period | -0.255 | 0.177 | -1.439 | 0.153 |

#### Fat Intake.

Multilevel longitudinal model; without physical activity covariates

|  | Estimate | SE | t-value | p-value |
| --- | --- | --- | --- | --- |
| (Intercept) | 3.005 | 0.152 | 19.772 | 0.000 |
| Treatment Period (vs. Baseline) | -0.392 | 0.162 | -2.418 | 0.017 |
| Deflated Condition X Treatment Period | 0.536 | 0.213 | 2.516 | 0.013 |
| Inflated Condition X Treatment Period | 0.248 | 0.226 | 1.099 | 0.274 |

Multilevel longitudinal model; with physical activity covariates

|  | Estimate | SE | t-value | p-value |
| --- | --- | --- | --- | --- |
| (Intercept) | 2.849 | 0.279 | 10.193 | 0.000 |
| Treatment Period (vs. Baseline) | -0.384 | 0.161 | -2.391 | 0.018 |
| Step Count | 0.000 | 0.000 | 0.452 | 0.651 |
| Other Physical Activities | 0.012 | 0.010 | 1.204 | 0.229 |
| Deflated Condition X Treatment Period | 0.535 | 0.214 | 2.505 | 0.014 |
| Inflated Condition X Treatment Period | 0.243 | 0.223 | 1.092 | 0.277 |

#### Sugar Intake.

Multilevel longitudinal model; without physical activity covariates

|  | Estimate | SE | t-value | p-value |
| --- | --- | --- | --- | --- |
| (Intercept) | 3.131 | 0.156 | 20.072 | 0.000 |
| Treatment Period (vs. Baseline) | -0.085 | 0.158 | -0.539 | 0.590 |
| Deflated Condition X Treatment Period | -0.028 | 0.217 | -0.128 | 0.899 |
| Inflated Condition X Treatment Period | -0.294 | 0.225 | -1.302 | 0.194 |

Multilevel longitudinal model; with physical activity covariates

|  | Estimate | SE | t-value | p-value |
| --- | --- | --- | --- | --- |
| (Intercept) | 3.081 | 0.282 | 10.936 | 0.000 |
| Treatment Period (vs. Baseline) | -0.088 | 0.158 | -0.554 | 0.580 |
| Step Count | 0.000 | 0.000 | 0.253 | 0.801 |
| Other Physical Activities | -0.001 | 0.010 | -0.131 | 0.896 |
| Deflated Condition X Treatment Period | -0.027 | 0.217 | -0.124 | 0.901 |
| Inflated Condition X Treatment Period | -0.290 | 0.226 | -1.286 | 0.200 |

#### Vegetable Intake.

Multilevel longitudinal model; without physical activity covariates

|  | Estimate | SE | t-value | p-value |
| --- | --- | --- | --- | --- |
| (Intercept) | 3.979 | 0.242 | 16.443 | 0.000 |
| Deflated Condition (vs. Accurate) | 0.646 | 0.321 | 2.012 | 0.046 |
| Inflated Condition (vs. Accurate) | 0.421 | 0.335 | 1.258 | 0.211 |
| Treatment Period (vs. Baseline) | 0.365 | 0.152 | 2.401 | 0.017 |
| Deflated Condition X Treatment Period | -0.567 | 0.193 | -2.934 | 0.004 |
| Inflated Condition X Treatment Period | -0.334 | 0.216 | -1.548 | 0.123 |

Multilevel longitudinal model; with physical activity covariates

|  | Estimate | SE | t-value | p-value |
| --- | --- | --- | --- | --- |
| (Intercept) | 3.708 | 0.291 | 12.722 | 0.000 |
| Deflated Condition (vs. Accurate) | 0.628 | 0.321 | 1.956 | 0.053 |
| Inflated Condition (vs. Accurate) | 0.387 | 0.336 | 1.151 | 0.252 |
| Treatment Period (vs. Baseline) | 0.355 | 0.154 | 2.309 | 0.022 |
| Step Count | 0.000 | 0.000 | 1.824 | 0.069 |
| Other Physical Activities | 0.002 | 0.008 | 0.235 | 0.814 |
| Deflated Condition X Treatment Period | -0.559 | 0.197 | -2.842 | 0.005 |
| Inflated Condition X Treatment Period | -0.314 | 0.219 | -1.432 | 0.153 |

#### Smoking.

Multilevel longitudinal model; without physical activity covariates

|  | Estimate | SE | t-value | p-value |
| --- | --- | --- | --- | --- |
| (Intercept) | 3.706 | 1.480 | 2.504 | 0.014 |
| Treatment Period (vs. Baseline) | -0.672 | 0.827 | -0.812 | 0.417 |
| Deflated Condition X Treatment Period | 1.701 | 1.362 | 1.248 | 0.213 |
| Inflated Condition X Treatment Period | -0.516 | 1.194 | -0.432 | 0.666 |

Multilevel longitudinal model; with physical activity covariates

|  | Estimate | SE | t-value | p-value |
| --- | --- | --- | --- | --- |
| (Intercept) | 4.974 | 2.375 | 2.246 | 0.025 |
| Treatment Period (vs. Baseline) | -0.618 | 1.322 | -0.769 | 0.442 |
| Step Count | 0.000 | 0.000 | -1.347 | 0.179 |
| Other Physical Activities | 0.020 | 0.086 | 0.594 | 0.553 |
| Deflated Condition X Treatment Period | 1.687 | 1.819 | 1.250 | 0.212 |
| Inflated Condition X Treatment Period | -0.608 | 1.824 | -0.515 | 0.607 |

#### Alcohol Consumption.

Multilevel longitudinal model; without physical activity covariates

|  | Estimate | SE | t-value | p-value |
| --- | --- | --- | --- | --- |
| (Intercept) | 3.660 | 0.620 | 5.900 | 0.000 |
| Treatment Period (vs. Baseline) | -0.093 | 0.348 | -0.267 | 0.790 |
| Deflated Condition X Treatment Period | -0.030 | 0.532 | -0.057 | 0.955 |
| Inflated Condition X Treatment Period | -1.355 | 0.671 | -2.019 | 0.046 |

Multilevel longitudinal model; with physical activity covariates

|  | Estimate | SE | t-value | p-value |
| --- | --- | --- | --- | --- |
| (Intercept) | 2.977 | 0.737 | 4.040 | 0.000 |
| Treatment Period (vs. Baseline) | -0.053 | 0.354 | -0.151 | 0.880 |
| Step Count | 0.000 | 0.000 | 0.677 | 0.499 |
| Other Physical Activities | 0.066 | 0.037 | 1.804 | 0.072 |
| Deflated Condition X Treatment Period | -0.021 | 0.523 | -0.040 | 0.969 |
| Inflated Condition X Treatment Period | -1.381 | 0.665 | -2.077 | 0.040 |

## Effects of Meta-Mindset Intervention

### Activity Adequacy Mindset

Multilevel longitudinal model; without physical activity covariates

|  | Estimate | SE | t-value | p-value |
| --- | --- | --- | --- | --- |
| (Intercept) | 3.453 | 0.122 | 28.222 | 0.000 |
| Treatment Period (vs. Baseline) | 0.037 | 0.149 | 0.247 | 0.806 |
| Meta Condition X Treatment Period | 0.581 | 0.185 | 3.133 | 0.002 |

Multilevel longitudinal model; with physical activity covariates

|  | Estimate | SE | t-value | p-value |
| --- | --- | --- | --- | --- |
| (Intercept) | 3.407 | 0.128 | 26.642 | 0.000 |
| Treatment Period (vs. Baseline) | -0.007 | 0.138 | -0.048 | 0.962 |
| Step Count | 0.240 | 0.066 | 3.649 | 0.000 |
| Other Physical Activities | 0.023 | 0.013 | 1.799 | 0.073 |
| Meta Condition X Treatment Period | 0.567 | 0.179 | 3.173 | 0.002 |

### Health Outcomes

#### Functional Health.

Multilevel longitudinal model; without physical activity covariates

|  | Estimate | SE | t-value | p-value |
| --- | --- | --- | --- | --- |
| (Intercept) | 4.462 | 0.061 | 72.714 | 0.000 |
| Treatment Period (vs. Baseline) | -0.110 | 0.047 | -2.324 | 0.022 |
| Meta Condition X Treatment Period | 0.182 | 0.070 | 2.602 | 0.011 |

Multilevel longitudinal model; with physical activity covariates

|  | Estimate | SE | t-value | p-value |
| --- | --- | --- | --- | --- |
| (Intercept) | 4.477 | 0.081 | 55.546 | 0.000 |
| Treatment Period (vs. Baseline) | -0.116 | 0.048 | -2.391 | 0.019 |
| Step Count | 0.062 | 0.044 | 1.419 | 0.158 |
| Other Physical Activities | -0.003 | 0.012 | -0.254 | 0.800 |
| Meta Condition X Treatment Period | 0.184 | 0.068 | 2.714 | 0.008 |

#### Mental Health.

Multilevel longitudinal model; without physical activity covariates

|  | Estimate | SE | t-value | p-value |
| --- | --- | --- | --- | --- |
| (Intercept) | 3.732 | 0.072 | 51.976 | 0.000 |
| Treatment Period (vs. Baseline) | 0.157 | 0.071 | 2.224 | 0.029 |
| Meta Condition X Treatment Period | 0.035 | 0.093 | 0.371 | 0.712 |

Multilevel longitudinal model; with physical activity covariates

|  | Estimate | SE | t-value | p-value |
| --- | --- | --- | --- | --- |
| (Intercept) | 3.704 | 0.080 | 46.118 | 0.000 |
| Treatment Period (vs. Baseline) | 0.160 | 0.071 | 2.263 | 0.026 |
| Step Count | -0.021 | 0.053 | -0.399 | 0.691 |
| Other Physical Activities | 0.007 | 0.012 | 0.540 | 0.590 |
| Meta Condition X Treatment Period | 0.029 | 0.094 | 0.304 | 0.762 |

#### Self-Esteem.

Multilevel longitudinal model; without physical activity covariates

|  | Estimate | SE | t-value | p-value |
| --- | --- | --- | --- | --- |
| (Intercept) | 3.793 | 0.099 | 38.434 | 0.000 |
| Treatment Period (vs. Baseline) | 0.102 | 0.058 | 1.776 | 0.079 |
| Meta Condition X Treatment Period | -0.132 | 0.106 | -1.245 | 0.216 |

Multilevel longitudinal model; with physical activity covariates

|  | Estimate | SE | t-value | p-value |
| --- | --- | --- | --- | --- |
| (Intercept) | 3.854 | 0.105 | 36.777 | 0.000 |
| Treatment Period (vs. Baseline) | 0.106 | 0.057 | 1.856 | 0.067 |
| Step Count | -0.063 | 0.066 | -0.949 | 0.344 |
| Other Physical Activities | -0.016 | 0.012 | -1.351 | 0.179 |
| Meta Condition X Treatment Period | -0.120 | 0.108 | -1.107 | 0.271 |

#### Bodyweight.

Multilevel longitudinal model; without physical activity covariates

|  | Estimate | SE | t-value | p-value |
| --- | --- | --- | --- | --- |
| (Intercept) | 161.878 | 5.235 | 30.921 | 0.000 |
| Treatment Period (vs. Baseline) | -0.059 | 0.421 | -0.139 | 0.890 |
| Meta Condition X Treatment Period | 0.522 | 0.700 | 0.745 | 0.458 |

Multilevel longitudinal model; with physical activity covariates

|  | Estimate | SE | t-value | p-value |
| --- | --- | --- | --- | --- |
| (Intercept) | 161.801 | 5.278 | 30.655 | 0.000 |
| Treatment Period (vs. Baseline) | -0.030 | 0.428 | -0.071 | 0.943 |
| Step Count | -0.289 | 0.411 | -0.703 | 0.484 |
| Other Physical Activities | 0.016 | 0.103 | 0.157 | 0.876 |
| Meta Condition X Treatment Period | 0.507 | 0.713 | 0.711 | 0.479 |

#### VO2 Max.

Multilevel longitudinal model; without physical activity covariates

|  | Estimate | SE | t-value | p-value |
| --- | --- | --- | --- | --- |
| (Intercept) | 31.859 | 0.916 | 34.789 | 0.000 |
| Treatment Period (vs. Baseline) | 0.547 | 0.307 | 1.780 | 0.079 |
| Meta Condition X Treatment Period | -0.331 | 0.460 | -0.719 | 0.474 |

Multilevel longitudinal model; with physical activity covariates

|  | Estimate | SE | t-value | p-value |
| --- | --- | --- | --- | --- |
| (Intercept) | 31.783 | 0.956 | 33.261 | 0.000 |
| Treatment Period (vs. Baseline) | 0.516 | 0.303 | 1.701 | 0.093 |
| Step Count | 0.368 | 0.289 | 1.272 | 0.207 |
| Other Physical Activities | 0.020 | 0.060 | 0.342 | 0.733 |
| Meta Condition X Treatment Period | -0.322 | 0.460 | -0.699 | 0.486 |

#### Mean Arterial Blood Pressure.

Multilevel longitudinal model; without physical activity covariates

|  | Estimate | SE | t-value | p-value |
| --- | --- | --- | --- | --- |
| (Intercept) | 92.577 | 1.551 | 59.684 | 0.000 |
| Treatment Period (vs. Baseline) | -2.301 | 1.552 | -1.482 | 0.142 |
| Meta Condition X Treatment Period | 3.520 | 1.821 | 1.933 | 0.056 |

Multilevel longitudinal model; with physical activity covariates

|  | Estimate | SE | t-value | p-value |
| --- | --- | --- | --- | --- |
| (Intercept) | 92.951 | 1.628 | 57.094 | 0.000 |
| Treatment Period (vs. Baseline) | -2.387 | 1.570 | -1.520 | 0.132 |
| Step Count | 0.839 | 0.962 | 0.872 | 0.385 |
| Other Physical Activities | -0.084 | 0.215 | -0.392 | 0.696 |
| Meta Condition X Treatment Period | 3.592 | 1.843 | 1.948 | 0.054 |

#### Resting Heart Rate.

Multilevel longitudinal model; without physical activity covariates

|  | Estimate | SE | t-value | p-value |
| --- | --- | --- | --- | --- |
| (Intercept) | 71.720 | 1.207 | 59.436 | 0.000 |
| Treatment Period (vs. Baseline) | 0.464 | 1.335 | 0.348 | 0.729 |
| Meta Condition X Treatment Period | 0.766 | 1.733 | 0.442 | 0.659 |

Multilevel longitudinal model; with physical activity covariates

|  | Estimate | SE | t-value | p-value |
| --- | --- | --- | --- | --- |
| (Intercept) | 72.293 | 1.410 | 51.278 | 0.000 |
| Treatment Period (vs. Baseline) | 0.480 | 1.321 | 0.363 | 0.717 |
| Step Count | -0.513 | 0.973 | -0.527 | 0.599 |
| Other Physical Activities | -0.148 | 0.139 | -1.065 | 0.289 |
| Meta Condition X Treatment Period | 0.892 | 1.718 | 0.519 | 0.605 |

### Affective Processes

#### Negative Affect.

Multilevel longitudinal model; without physical activity covariates

|  | Estimate | SE | t-value | p-value |
| --- | --- | --- | --- | --- |
| (Intercept) | 2.145 | 0.065 | 32.882 | 0.000 |
| Treatment Period (vs. Baseline) | -0.092 | 0.054 | -1.707 | 0.091 |
| Meta Condition X Treatment Period | -0.133 | 0.073 | -1.831 | 0.071 |

Multilevel longitudinal model; with physical activity covariates

|  | Estimate | SE | t-value | p-value |
| --- | --- | --- | --- | --- |
| (Intercept) | 2.153 | 0.069 | 31.316 | 0.000 |
| Treatment Period (vs. Baseline) | -0.089 | 0.055 | -1.624 | 0.108 |
| Step Count | -0.022 | 0.025 | -0.889 | 0.374 |
| Other Physical Activities | -0.003 | 0.003 | -1.034 | 0.302 |
| Meta Condition X Treatment Period | -0.131 | 0.072 | -1.818 | 0.073 |

#### Positive Affect.

Multilevel longitudinal model; without physical activity covariates

|  | Estimate | SE | t-value | p-value |
| --- | --- | --- | --- | --- |
| (Intercept) | 2.870 | 0.062 | 46.365 | 0.000 |
| Treatment Period (vs. Baseline) | -0.305 | 0.056 | -5.429 | 0.000 |
| Meta Condition X Treatment Period | 0.237 | 0.096 | 2.465 | 0.016 |

Multilevel longitudinal model; with physical activity covariates

|  | Estimate | SE | t-value | p-value |
| --- | --- | --- | --- | --- |
| (Intercept) | 2.844 | 0.061 | 46.978 | 0.000 |
| Treatment Period (vs. Baseline) | -0.296 | 0.057 | -5.205 | 0.000 |
| Step Count | -0.003 | 0.032 | -0.101 | 0.920 |
| Other Physical Activities | 0.006 | 0.004 | 1.539 | 0.124 |
| Meta Condition X Treatment Period | 0.225 | 0.097 | 2.314 | 0.023 |

### Behavioral Processes

#### Step Count.

Multilevel longitudinal model; without physical activity covariates

|  | Estimate | SE | t-value | p-value |
| --- | --- | --- | --- | --- |
| (Intercept) | 6859.670 | 254.427 | 26.961 | 0.000 |
| Treatment Period (vs. Baseline) | 182.152 | 256.470 | 0.710 | 0.479 |
| Meta Condition X Treatment Period | 15.594 | 337.586 | 0.046 | 0.963 |

#### Other Physical Activities.

Multilevel longitudinal model; without physical activity covariates

|  | Estimate | SE | t-value | p-value |
| --- | --- | --- | --- | --- |
| (Intercept) | 4.470 | 0.623 | 7.180 | 0.000 |
| Treatment Period (vs. Baseline) | -0.905 | 0.604 | -1.498 | 0.136 |
| Meta Condition X Treatment Period | 1.643 | 0.951 | 1.728 | 0.087 |

#### Overeating.

Multilevel longitudinal model; without physical activity covariates

|  | Estimate | SE | t-value | p-value |
| --- | --- | --- | --- | --- |
| (Intercept) | 1.061 | 0.103 | 10.347 | 0.000 |
| Treatment Period (vs. Baseline) | -0.138 | 0.086 | -1.598 | 0.113 |
| Meta Condition X Treatment Period | -0.038 | 0.115 | -0.329 | 0.743 |

Multilevel longitudinal model; with physical activity covariates

|  | Estimate | SE | t-value | p-value |
| --- | --- | --- | --- | --- |
| (Intercept) | 1.068 | 0.109 | 9.796 | 0.000 |
| Treatment Period (vs. Baseline) | -0.146 | 0.087 | -1.680 | 0.096 |
| Step Count | -0.045 | 0.040 | -1.109 | 0.268 |
| Other Physical Activities | -0.001 | 0.007 | -0.101 | 0.919 |
| Meta Condition X Treatment Period | -0.030 | 0.114 | -0.260 | 0.796 |

#### Fat Intake.

Multilevel longitudinal model; without physical activity covariates

|  | Estimate | SE | t-value | p-value |
| --- | --- | --- | --- | --- |
| (Intercept) | 2.866 | 0.177 | 16.235 | 0.000 |
| Treatment Period (vs. Baseline) | -0.146 | 0.185 | -0.789 | 0.432 |
| Meta Condition X Treatment Period | -0.051 | 0.223 | -0.227 | 0.821 |

Multilevel longitudinal model; with physical activity covariates

|  | Estimate | SE | t-value | p-value |
| --- | --- | --- | --- | --- |
| (Intercept) | 2.838 | 0.183 | 15.479 | 0.000 |
| Treatment Period (vs. Baseline) | -0.161 | 0.189 | -0.856 | 0.394 |
| Step Count | -0.010 | 0.058 | -0.163 | 0.870 |
| Other Physical Activities | 0.010 | 0.011 | 0.894 | 0.372 |
| Meta Condition X Treatment Period | -0.048 | 0.227 | -0.214 | 0.831 |

#### Sugar Intake.

Multilevel longitudinal model; without physical activity covariates

|  | Estimate | SE | t-value | p-value |
| --- | --- | --- | --- | --- |
| (Intercept) | 2.988 | 0.164 | 18.211 | 0.000 |
| Treatment Period (vs. Baseline) | -0.192 | 0.166 | -1.162 | 0.249 |
| Meta Condition X Treatment Period | 0.215 | 0.212 | 1.017 | 0.312 |

Multilevel longitudinal model; with physical activity covariates

|  | Estimate | SE | t-value | p-value |
| --- | --- | --- | --- | --- |
| (Intercept) | 3.003 | 0.168 | 17.840 | 0.000 |
| Treatment Period (vs. Baseline) | -0.167 | 0.166 | -1.004 | 0.318 |
| Step Count | 0.036 | 0.069 | 0.520 | 0.603 |
| Other Physical Activities | -0.008 | 0.008 | -1.000 | 0.318 |
| Meta Condition X Treatment Period | 0.199 | 0.211 | 0.946 | 0.347 |

#### Vegetable Intake.

Multilevel longitudinal model; without physical activity covariates

|  | Estimate | SE | t-value | p-value |
| --- | --- | --- | --- | --- |
| (Intercept) | 4.268 | 0.174 | 24.499 | 0.000 |
| Treatment Period (vs. Baseline) | 0.071 | 0.157 | 0.454 | 0.651 |
| Meta Condition X Treatment Period | 0.003 | 0.197 | 0.016 | 0.987 |

Multilevel longitudinal model; with physical activity covariates

|  | Estimate | SE | t-value | p-value |
| --- | --- | --- | --- | --- |
| (Intercept) | 4.250 | 0.182 | 23.403 | 0.000 |
| Treatment Period (vs. Baseline) | 0.127 | 0.146 | 0.869 | 0.387 |
| Step Count | 0.086 | 0.058 | 1.473 | 0.142 |
| Other Physical Activities | -0.004 | 0.013 | -0.285 | 0.776 |
| Meta Condition X Treatment Period | -0.042 | 0.189 | -0.223 | 0.824 |

#### Smoking.

Multilevel longitudinal model; without physical activity covariates

|  | Estimate | SE | t-value | p-value |
| --- | --- | --- | --- | --- |
| (Intercept) | 3.268 | 1.622 | 2.014 | 0.047 |
| Treatment Period (vs. Baseline) | 0.443 | 0.534 | 0.829 | 0.409 |
| Meta Condition X Treatment Period | 0.450 | 2.385 | 0.189 | 0.851 |

Multilevel longitudinal model; with physical activity covariates

|  | Estimate | SE | t-value | p-value |
| --- | --- | --- | --- | --- |
| (Intercept) | 3.198 | 1.619 | 1.976 | 0.051 |
| Treatment Period (vs. Baseline) | 0.333 | 0.540 | 0.616 | 0.539 |
| Step Count | 0.977 | 0.634 | 1.542 | 0.124 |
| Other Physical Activities | 0.036 | 0.066 | 0.552 | 0.581 |
| Meta Condition X Treatment Period | 0.492 | 2.353 | 0.209 | 0.835 |

#### Alcohol Consumption.

Multilevel longitudinal model; without physical activity covariates

|  | Estimate | SE | t-value | p-value |
| --- | --- | --- | --- | --- |
| (Intercept) | 3.826 | 0.662 | 5.776 | 0.000 |
| Treatment Period (vs. Baseline) | 0.333 | 0.411 | 0.810 | 0.420 |
| Meta Condition X Treatment Period | -0.459 | 0.594 | -0.772 | 0.442 |

Multilevel longitudinal model; with physical activity covariates

|  | Estimate | SE | t-value | p-value |
| --- | --- | --- | --- | --- |
| (Intercept) | 3.488 | 0.654 | 5.336 | 0.000 |
| Treatment Period (vs. Baseline) | 0.328 | 0.433 | 0.758 | 0.451 |
| Step Count | 0.016 | 0.128 | 0.124 | 0.901 |
| Other Physical Activities | 0.091 | 0.055 | 1.636 | 0.103 |
| Meta Condition X Treatment Period | -0.535 | 0.609 | -0.878 | 0.382 |

# Appendix

This Appendix includes:

- Recruitment materials
- Pre-screen survey
- Onboarding materials
- Onboarding session introductory script for experimenter
- Onboarding procedures (illustrations)
- Meta-mindset intervention materials and reflection activities

Complete data sets and R scripts containing data processing and statistical analyses will be made available on Open Science Framework (https://osf.io/8ea5r/?view_only=35003df8eb984ae888547a9eab27eb21).

## Recruitment Materials

**Figure S1.** Research participant recruitment flyer.

**Pre-screen Survey**

[Eligible responses marked in yellow]

Thank you for your interest in participating in the wearable tech study!

Do you use an iPhone 5 S or newer?

- Yes (1)
- No (2)

Do you have a security software on your iPhone (e.g., through your employer), that requires you to have a lock code?

- Yes (1)
- Not sure (2)
- No (3)

Over the past six months, on average how many times per week did you WALK for at least 10 minutes at a time?

- 0 (0)
- 1 (1)
- 2 (2)
- 3-4 (3)
- 5 or more (4)

Over the past six months, on average how many times per week did you exercise OTHER THAN WALKING?

- 0 (0)
- 1 (1)
- 2 (2)
- 3-4 (3)
- 5 or more (4)

How often do you use a device (a wearable device or phone app) to track steps or other forms of physical activity?

- twice a week or more (5)
- once a week (4)
- 2-3 times per month (3)
- once a month (2)
- less than once a month (1)
- never (0)

Are you able to commit to a 5-week long study? 
You will need to attend two 1.5-hour sessions on Stanford campus in the beginning and end of the 5-week period, consistently wear the Apple Watch throughout the 5-week period complete brief daily surveys. You can sign up for a 5-week period sometime between January - March 2019 [adapted to current date].

- Yes (1)
- No (0)

Has your doctor ever said that you have a heart condition and that you should only do physical activity recommended by a doctor?

- Yes (1)
- No (0)

Heart and chest pain Do you feel pain in your chest when you do physical activity?

- Yes (1)
- No (0)

In the past month, have you had chest pain when you were not doing physical activity?

- Yes (1)
- No (0)

Do you lose your balance because of dizziness or do you ever lose consciousness?

- Yes (1)
- No (0)

Do you have a bone or joint problem that could be made worse by a change in your physical activity?

- Yes (1)
- No (0)

Is your doctor currently prescribing drugs (for example, water pills) for your blood pressure or heart condition?

- Yes (1)
- No (0)

Do you know of any other reason why you should not do physical activity?

- Yes (1)
- No (0)

Are you pregnant?

- Yes (1)
- No (0)

How old are you? __________

What gender do you identify with?

- Female (1)
- Male (2)
- Other (3)

Employment Status: Are you currently…?

- Employed for wages (1)
- Self-employed (2)
- Out of work and looking for work (3)
- Out of work but not currently looking for work (4)
- A homemaker (5)
- A student (6)
- Military (7)
- Retired (8)
- Unable to work (9)

Are you a US American citizen?

- Yes (1)
- No (2)

What is your English language proficiency?

- Basic (1)
- Intermediate (2)
- Proficient (3)
- Native speaker (4)

Finally, please leave your contact information so we can get in touch if you qualify.

What is your email address?_________

What is your phone number (optional)? __________

## Onboarding Materials

**Figure S2.** Information packet given to participants in the onboarding session, including instructions for participation; information on interacting with the Apple Watch; a primer on the health benefits of walking (with references to scientific research); and consent form.

## Onboarding Session Introductory Script for Experimenter

[Intro]

Hello, are you here for the Appel Watch study? What’s your name?

Hi [name], thank you for coming in to participate! How are you doing today? My name is [Kris/ Octavia], and I’m going to get you started with the study today.

First, I will explain the details of the study and get your consent to participate.

Then, you will complete a survey, a cognitive task, and a fitness task.

All of this is going to take up to 75 minutes. Sound good?

[Start pairing process]

Ok, first, let’s pair your phone with a watch. Did you download the latest iPhone software?

*[Go through “Pairing” steps in* [*Watch setup protocol*](https://docs.google.com/document/d/1mpXyhFqCYtPwFDPDTXFOuNXCxKFtiXgSU-mYC36P2JY/edit?usp=sharing)*].* Ok, while this is syncing, let’s talk about the details of the study.

[Study Overview]

Our team does a lot of research on wearable technology, and over the past few years we’ve been developing wearables that track physical activity more accurately. We are doing this study to test our new step count tracker and how users experience it.  *[Important to state same way every time]*

We are now going to talk through the details of the study. There’s a lot of information, but don’t worry, all of it is also on these handouts *[show folder]* that you will get to take home.

First, we are going to go through the consent form *[take out* [*consent form*](https://docs.google.com/document/d/1JBP6fGeXwrP0PcX7q2SEufbVSigRvfDcD3_EalbCzdo/edit?usp=sharing) *and point to the relevant sections while talking]*.

[Consent Form: Description]

In this study, we ask you to wear your Apple Watch every day from wake to sleep for 5 weeks to track your activity levels.

You will also be asked to complete a number of surveys.

In this onboarding session, you will complete the first survey which takes about 15 minutes. It asks about your lifestyle, well-being, attitudes about health, and personal background. Some of these questions can feel personal, but be assured that the surveys are completely confidential and anonymous. They will not be linked to your name, but simply to an anonymous participant ID which is located here *[point at ID on handout cover sheet]*.

In addition to this onboarding survey, you will also complete short 1-2 minute surveys each evening. All of these surveys ask similar questions so they may seem repetitive, but this allows us to track how your experience develops over time. Please answer all of the questions mindfully and honestly.

Just once a week, you will receive longer weekly surveys instead of your quick daily check-in. These surveys take about 15 minutes, so just be prepared for that and make sure you have sufficient time to complete the survey. Today is *[x day of the week],* so your weekly surveys will come every *[that day of the week]* for the coming weeks.

Today, we will also take some physiological measures (such as weight and blood pressure), and you will do a short fitness test. You will also complete a brief cognitive task.

When you come back for your offboarding session in the end of the study, we will collect the same measures again.

Finally, we will collect your credit card information -- this is just to ensure that you return the Watch in the end of the study, and as long as you do that there will be no charges. *[if asked, say they will be charged the price of the watch = $150]*

[Consent Form: Time Involvement]

This study will last 5 weeks.

[Consent Form: Risks and Benefits]

There is minimal risk involved. We guarantee no benefits, although we hope you will find the study interesting and enjoyable.

[Consent Form: Confidentiality]

Again, we will protect your data and keep it confidential and anonymous.

[Consent Form: Payments]

You will receive up to $175 for your participation in the study, depending on your participation levels. 100% participation includes attending onboarding/offboarding sessions, wearing watch daily whenever you’re awake, and responding to all surveys. *[if asked, here are details: baseline payment for wearing the device consistently, cooperating if we reach out to them, and attending all in-lab sessions = $100; completing at least 90% of questionnaires = $75]*

[Consent Form: Participant’s Rights]

Your participation is voluntary and you can withdraw at any point if you need to. *[if asked: if they withdraw, they will receive a prorated payment.]*

Do you have any questions? Please go ahead and sign here if you consent to participate. There is also a spare copy of this consent form in your folder to take home.

[Dos and Don’ts]

Now we will go over some dos and don’ts for this study. *[take out* [*handout*](https://docs.google.com/document/d/1A5Pp4o-r2rt62yzPPohdvxAtzTLdaaW1BpKaulwnlI0/edit?usp=sharing) *and go through the ‘what we ask of you’ section while talking]*.

Do wear your watch every day from wake to sleep. You should wear it snug on top of your wrist so that it’s close to your skin.

Do charge your phone and watch every night.

We suggest that you put the watch charger next to your bed, and when you go to bed put the watch on the charger like this *[demonstrate, show how it’s magnetic and the visual signal that it’s starting to charge]*. When you get up in the morning, put it on.

Be sure to take the watch off when you shower or go swimming -- it is water resistant but not waterproof *[don’t mention it’s Apple Watch 1]*

Do complete the daily and weekly surveys. You will be getting a text message at 8pm every night, and a reminder at 9pm. You just have to click on the link to get to the survey. You don’t have to fill out the survey right at 8pm, just make sure you fill it out the same evening that you get it, before midnight. And then just keep wearing the watch until you go to sleep. *[if participant says they can’t do it on a particular evening, tell them it’s fine to do it the next morning as an exception]*

Do make sure that your phone and watch are connected via bluetooth and that your phone is connected to the internet whenever possible

And be sure to come in for your offboarding session at the end of the 5 weeks.

Now here are some key things not to do throughout the study:

Don’t use any other activity, fitness, or health apps on your watch, your phone, or any other device. Doing so could interfere with the programming of our app. Also, it is really important for this study that you immerse yourself fully in the experience of our watch app.

Also, don’t change your watch face. This is the watch face you will be seeing *[show on own watch]*, and you shouldn’t change how it looks.

Don’t turn the watch on Airplane Mode or Do Not Disturb, and don’t turn off the watch. If you absolutely have to (for example, you go on an airplane or have an important test), just make sure you turn them back off afterwards. Here is how you do it *[demonstrate on own watch]*

Don’t do a software update on your watch or your phone at any point during study

Don’t add a passcode to your watch

And finally, don’t eat, drink coffee, or exercise 2 hours prior to your offboarding session

Do you have any questions?

[Interacting with your watch]

And now, just a few points on what to expect interacting with your watch. *[Talk through ‘interacting with your watch’ section on p.2 of handout]*

You will not see your step count during the first 7 days— the app will be busy calibrating to how you move! *[point to calibrating watch on handout]*

Your step count will appear a week from tomorrow like this *[point to step count on watch on handout]*

Your step count will update about every 15 minutes (to save battery life), so don’t be surprised if you don’t see the count updating immediately after you take a few steps

You will also get notifications every 2 hours updating you about your current step count *[point to watch with notification on handout]*

Besides the tracker, you will have access to a variety of basic watch features. This includes the calendar, messages, and heart rate which will be directly on your watch face *[point to these apps on watch on handout].*

Would you like to receive alerts for your messages and phone calls on your watch? Or just on your phone? [if they don’t get it, you can mention that some people like these alerts, but some find them annoying. Depending on answer, set notifications later during watch setup.]

And there are also other apps such as music, timer, and phone which you can access by pressing the crown *[show on own watch]*, and you can get back to the watch face by pressing the crown again *[show on own watch].* But again, don’t use any other fitness or health apps, including the ones that are there by Apple’s defaults.

Also, just so you know, we may contact you throughout the study to make sure the Watch is working ok.

[Benefits of walking]

Finally, here is a handout outlining some of the benefits you can get from walking *[turn page to Benefits handout and point]*

You may have heard people say that you need a certain number of steps per day to be healthy, but actually, the science shows that you can get health benefits at various amounts of steps

For example, walking 1,000 steps a day can reduce stress, and walking 2,000 steps a day can lower your mortality risk by 14%. As you can see, these benefits keep going up as you take more and more steps -- so for example at 15,000 steps a day, you can lower your mortality risk by 30%

But I really want to point out that the Apple Watch is meant to facilitate whatever health and fitness goals you have. You don’t have to change your behavior at all to be part of this study, but, if you want to try to get more steps each day, you are welcome to do so.

Do you have any questions?

Ok, well thank you again for helping us out with this study! We will now get you started on the survey *[hand them iPad]* -- you can go sit over there on the couch to fill it out. The survey should take about 15 - 20 minutes. In the end of that survey, you will be asked to register your phone number to receive your daily surveys. Let me know if you have any questions. *[Have them take their handout with them]*.

In the meantime, I will finish setting up your watch and phone. *[They may need to unlock it.]*

## Onboarding Procedures (Illustrations)

**Figure S3.** Photographs illustrating experimental procedures during the onboarding session.

**Figure S3a.** Introductory conversation between experimenter (left) and participant.


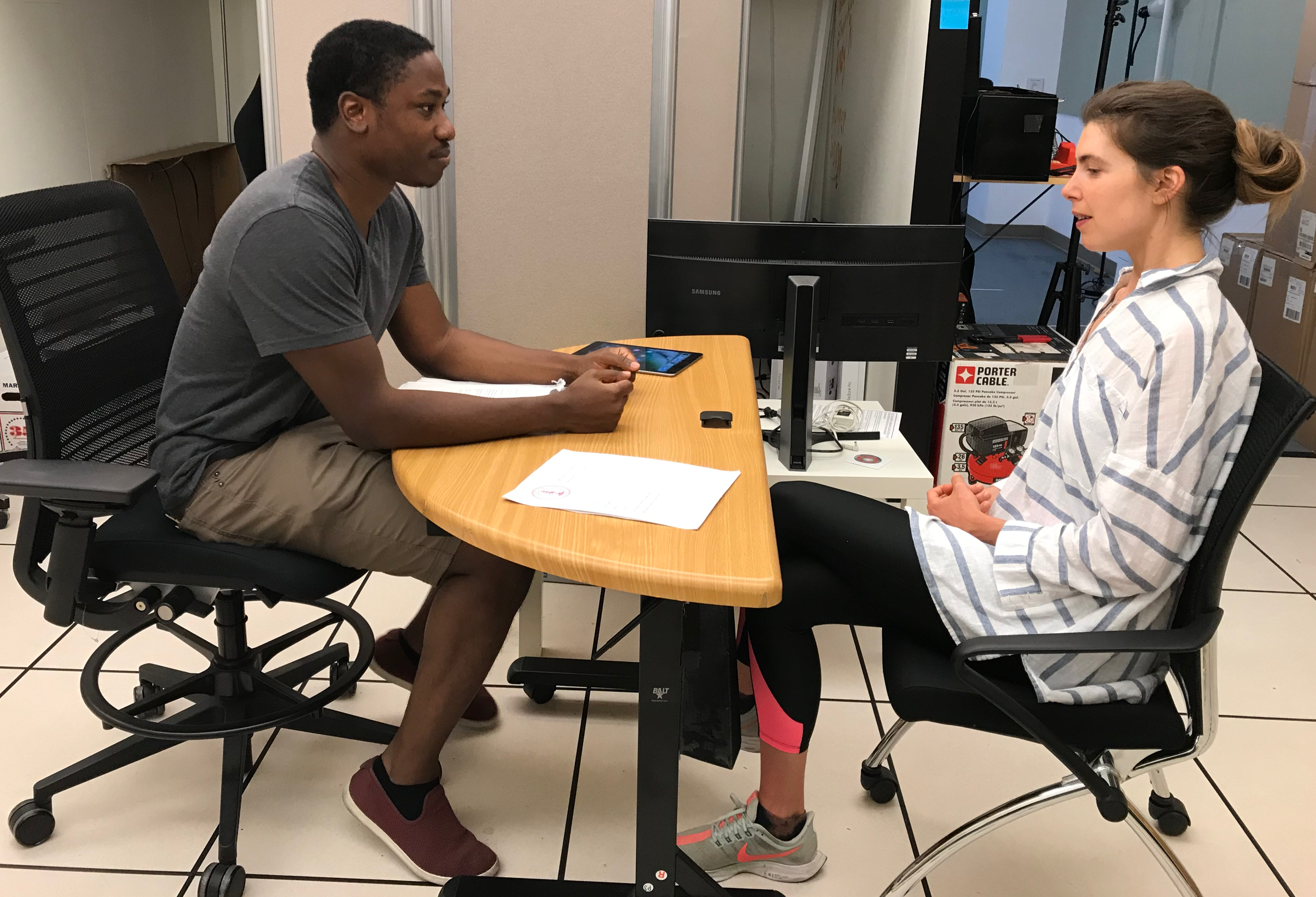


**Figure S3b.** Participant completing onboarding survey on iPad.


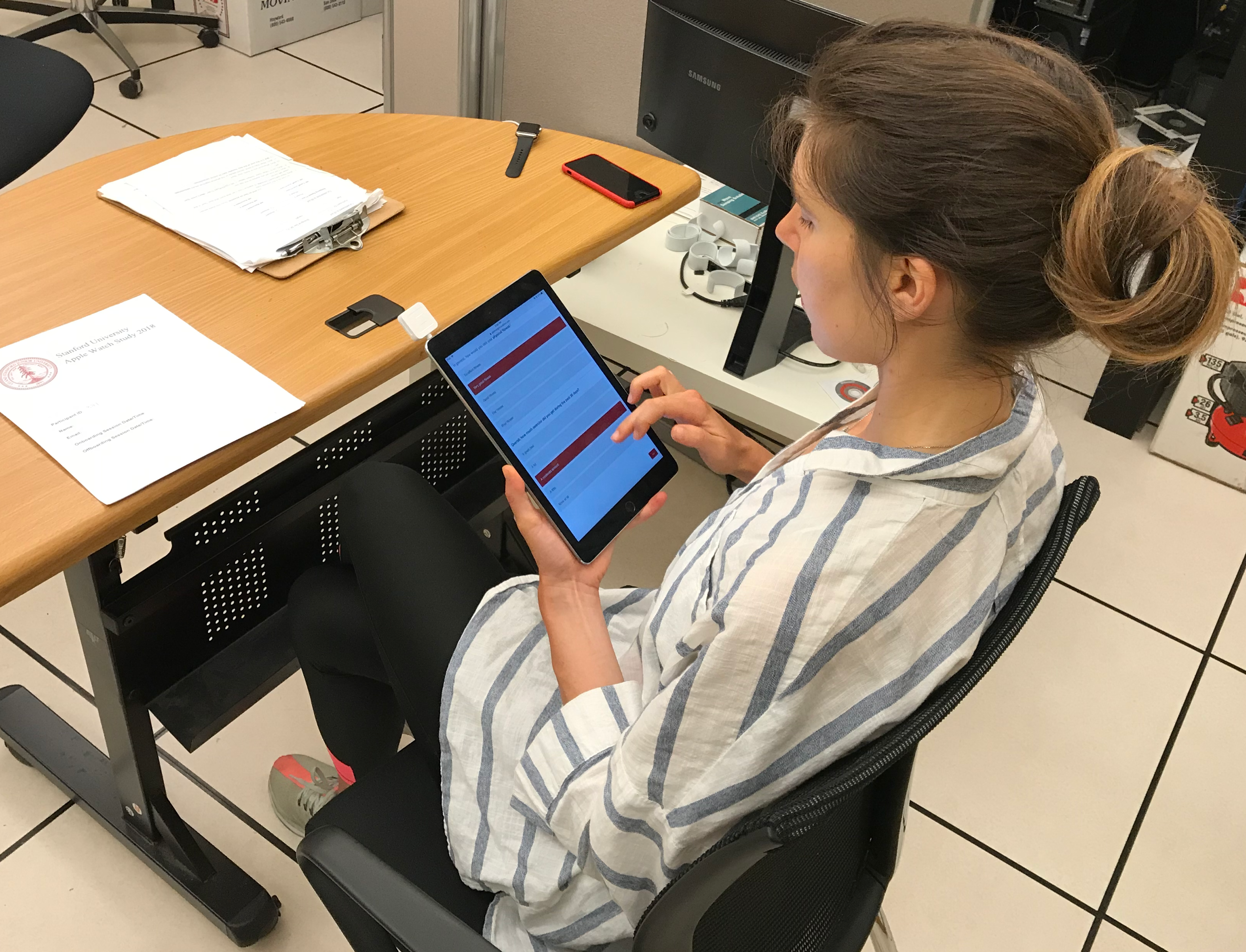


**Figure S3c.** Experimenter measuring participant’s blood pressure.


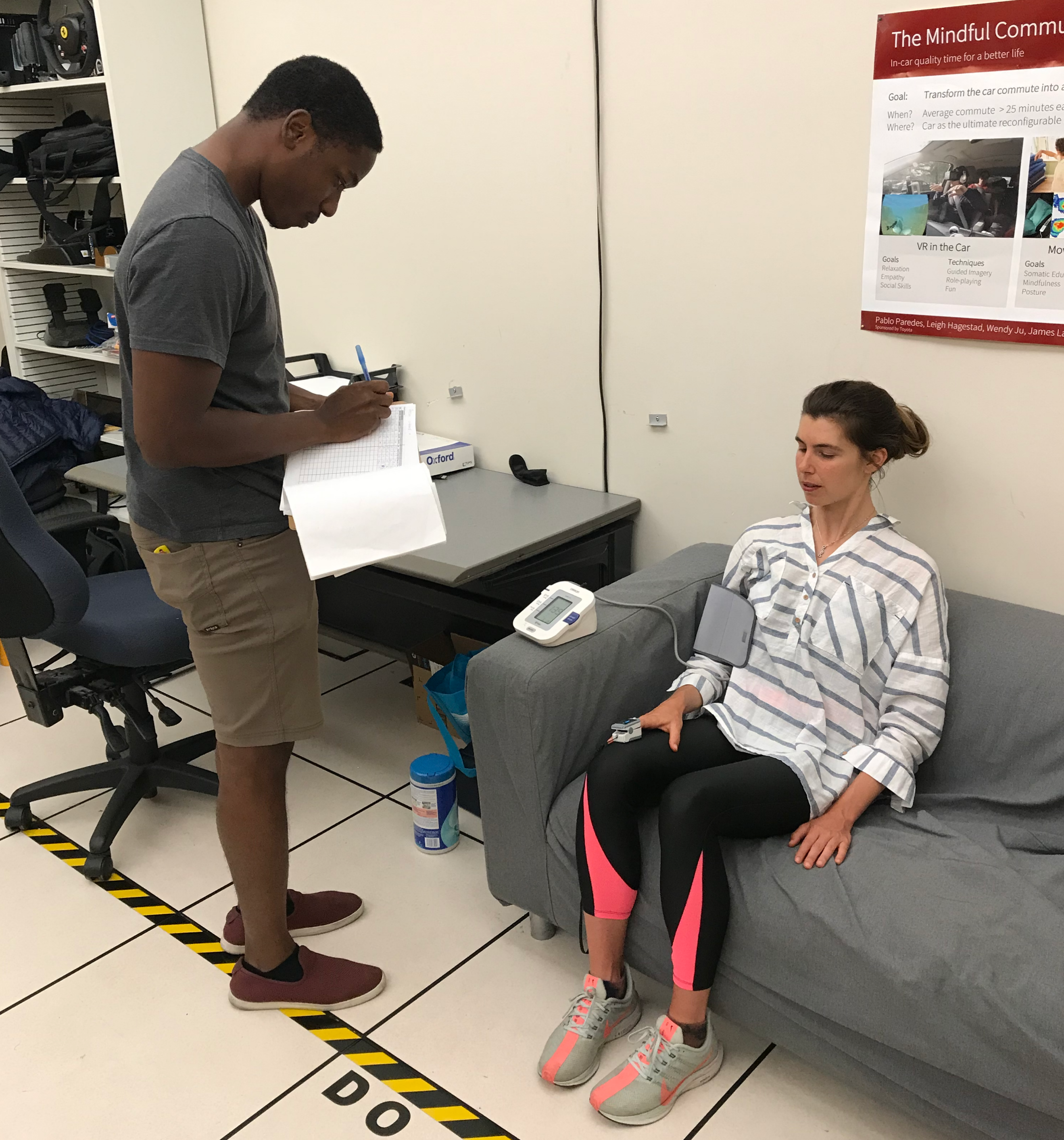


**Figure S3d.** Participant completing aerobic fitness test.

## Meta Mindset Intervention Materials and Reflection Activities

### Main Intervention (Administered in the first weekly survey via the Qualtrics online survey platform.)

Welcome! 

This is your first weekly survey. In this first weekly survey only, we will start you off with some more information on exercise that we think will benefit you. **Therefore, this first weekly survey will take about 20 - 25 min** (the usual weekly surveys will just take ~ 15 min). Please make sure you have sufficient time right now to complete this survey. **If it's more convenient, you can also come back to this survey later tonight** (just come back to this window or click the survey link in your text message again).
Thank you!

- Got it.

Later in this survey, you will watch a series of **3 videos explaining the power of "mindsets"** about exercise, which can have important effects on your wellbeing.
To make sure the videos display correctly, **please turn your phone sideways (horizontal) right now and ensure that your screen rotates**.
If the screen does not rotate, please do the following steps […]

Thanks!

- Got it, my screen is rotating sideways!

[ Comprehensive physical activity self-report measure comes here ]

Now, please think about your current level of exercise. Are you getting enough exercise? Please indicate generally how much exercise you get on a day-to-day basis.

- more than enough
- just enough
- not enough

You will now watch 3 videos. To make sure the videos display correctly, **please turn your phone sideways (horizontal) right now.**

First, you will watch a video explaining what a "mindset" is. The video is 2:45 min long. The red button to continue will appear in the lower right corner after you finish watching the video.

[ Video 1, What is a Mindset: <https://vimeo.com/295293381/5d47823036>, is displayed here ]

**What are features of mindsets?** Please check all of the following features that apply.

- Every person has mindsets
- Mindsets are simplified versions of reality
- Mindsets can shape our wellbeing, health, and behavior
- Mindsets are influenced by our culture
- Mindsets can create self-fulfilling prophecies
- Mindsets don’t matter at all

In case you were wondering about the answer to the question you just answered, mindsets have many of the features that were listed. Specifically, mindsets have the following features:

- Every person has mindsets
- Mindsets are simplified versions of reality
- Mindsets can shape our wellbeing, health, and behavior
- Mindsets are influenced by our culture
- Mindsets can create self-fulfilling prophecies

Next, you will watch a video about "exercise mindsets" specifically. The video is 4 min long. The red button to continue will appear in the lower right corner after you finish watching the video.

[ Video 2, Mindsets and Exercise: <https://vimeo.com/295294511/db5cb8f843>, is displayed here ]

Earlier, you answered a question about how much exercise you get on a daily basis. You answered that you get [inserted from participant’s answer above: not enough/ just enough/ more than enough] exercise. This is part of your current exercise mindset.

You just learned that your mindsets can be self-fulfilling. This means that your mindset about how much exercise you are getting can shape your health. 

**In light of this fact, how do you think your current exercise mindset might be influencing your health and wellbeing?**

- My current mindset promotes my health and wellbeing.
- My current mindset does not influence my health and wellbeing.
- My current mindset harms my health and wellbeing.

What led you to have the mindset that you get [not enough/ just enough/ more than enough] exercise? (Please check all that apply.)

- I did not count everyday activities as good exercise.
- I compared myself with people who get more exercise than me.
- I measured myself against exercise guidelines or targets that I didn't meet.
- I thought about how much less exercise I now get than I used to.
- Other (please specify)

Finally, you will watch a video about how mindsets can have self-fulfilling effects on health and wellbeing. This last video is 4:27 min long. The red button to continue will appear in the lower right corner after you finish watching the video.

[ Video 3, How Mindsets Work: <https://vimeo.com/295448930/024ff1c8e4>, is displayed here ]

How can mindsets and beliefs in your head affect your health and wellbeing? Please check all of the following pathways that apply.

- Mindsets can change what we notice and how we experience everyday life
- Mindsets can change our motivation to take care of our health
- Mindsets can influence how we feel (e.g., anxious or relaxed)
- Mindsets can activate or deactivate our immune system and other bodily systems
- None of the above

Great job! In fact, mindsets can influence our health and wellbeing in all of the following ways:

- Mindsets can change what we notice and how we experience everyday life
- Mindsets can change our motivation to take care of our health
- Mindsets can influence how we feel (e.g., anxious or relaxed)
- Mindsets can activate or deactivate our immune system and other bodily systems

As you just learned, having the mindset that you are getting enough exercise is useful. No matter how much exercise you are currently getting, there are strategies we can use to adopt a more helpful exercise mindset.

Below is a strategy to help you maintain or improve your exercise mindset in 3 simple steps — **Notice, Include & Celebrate!**

**1. Notice**

Notice any activities you did during the last 7 days that took physical effort.

These can be activities that you might not usually think of as exercise, for example:

- Walking
- Lifting shopping bags or other loads
- Carrying children
- Doing house or yard work
- Pushing carts or other objects

Note some of the activities you did in the last 7 days:

________________________________________________________________

________________________________________________________________

________________________________________________________________

________________________________________________________________

Great!  Your Apple Watch will start showing your step count tomorrow.  This will help you *notice* how much exercise you are getting just by going about your everyday activities

**2. Include**

Overall, how much exercise did you get during the last 7 days?   

But wait!  Before you answer this, be sure to include

- Any walking
- Other activities that you might previously not have considered as exercise (as noticed in the last step)
- A great deal
- A lot
- A moderate amount
- A little
- None at all

**3. Celebrate**

Now, pause and take a moment to *celebrate* yourself for the exercise you did during the last 7 days, and appreciate the benefits of this exercise. 
Here are some example benefits you may experience in the long term:

- Weight loss
- Lower blood pressure
- Protection from heart disease, diabetes, cancer

Here are some benefits you may experience immediately. Please check all benefits that you experienced during the last 7 days:

- Better mood
- Feeling more calm and relaxed
- Interacting with other people
- Fun and enjoyment
- Feeling refreshed, energized
- Overall good feeling in body
- More focus or creativity
- Feeling pride or gratitude
- Better sleep
- Less pain
- Experiencing a novel or beautiful environment
- Other (please specify)

### Booster Intervention (shortened reflection activities included in the daily check-ins via the Qualtrics online survey platform.)

**Welcome to your daily survey!**

As you now know, having the mindset that you are getting enough exercise can boost the benefits that your get from your exercise.

Below is your daily opportunity to practice having a positive exercise mindset in 3 simple steps — **Notice, Include & Celebrate!**

**1. Notice**

How many steps did you get today (as indicated by your Apple Watch)?

________________________________________________________________

**Notice any other activities you did today that took physical effort.**

These can be activities that you might not usually think of as exercise, or that are not counted in the Apple Watch step count.

Check any of the activities you did today, if any:

▢ lifting shopping bags, children, or other loads

▢ pushing carts or other objects

▢ house work

▢ yard work

▢ cooking or standing for extended periods of time

▢ playing with children or animals

▢ other (please specify) ________________________________________________

**2. Include**

**Overall, how much exercise did you get today?**

But wait! Before you answer this, be sure to include

- The steps you got today
- Other activities that you might previously not have considered as exercise (as noticed in the last step)
- Other activities not reflected by your Apple Watch step count (as noticed in the last step)
- A great deal
- A lot
- A moderate amount
- A little
- None at all

**3. Celebrate**

Now, take a moment to celebrate yourself for the exercise you did today, and appreciate the benefits of this exercise.

## Offboarding Session Experimenter Questions Regarding Suspicions

We want to ask you one more time if you used or looked at any other devices or apps to track your step count, other physical activity, or health since you started the study. Of course, there won’t be any penalty if you did look at other apps and devices, we just need to account for that in our analyses, so we need you to be completely honest.

- - *Yes, they looked at other apps/ devices*
  - *No, they did not.*

What did you think the study was about? …………………………………………………………………………………………

Was there anything in the study that you wondered about? *(Let them answer freely first.)* …………………………………………………………………………………………

*Then follow up:*

For example, were you wondering about any of the survey questions?

… about the Apple Watch or the step count app?

… why you were not supposed to look at other activity trackers?

……………………………………………………………………………………………

*(If they mention suspicions about the validity of the app or why they couldn’t look at other sources of step count information, why they were asked not to look at other health apps or step trackers, or that it was a psychological experiment:)*

What made you think that the step count might not be accurate?

What made you think the experiment might not be aimed at developing new technology?

What made you suspect this was a psychological experiment?

What made you think [whatever their suspicion is]?

……………………………………………………………………………………………

*After the interview, the research assistant assigned a rating of how suspicious participant was about study purpose/ deception, and of whether participant seemed to be lying regarding adhering to protocol/ not looking at other trackers, etc. (1 – not at all; 2 – slightly; 3 – somewhat; 4 – very; 5 – extremely; please explain …...……………...……)*

1. This study was pre-registered on ClinicalTrials.gov [49] [↑](#footnote-ref-2)
2. The experience sampling platform SurveySignal [56] was used to program the survey schedule and automatically send text messages. [↑](#footnote-ref-3)
3. Complete data sets, R scripts containing all data processing and statistical analyses will be made available on Open Science Framework (https://osf.io/8ea5r/?view_only=35003df8eb984ae888547a9eab27eb21) [↑](#footnote-ref-4)
